# An Overexpression Screen of *Toxoplasma gondii* Rab-GTPases Reveals Distinct Transport Routes to the Micronemes

**DOI:** 10.1371/journal.ppat.1003213

**Published:** 2013-03-07

**Authors:** Katrin Kremer, Dirk Kamin, Eva Rittweger, Jonathan Wilkes, Halley Flammer, Sabine Mahler, Joanne Heng, Christopher J. Tonkin, Gordon Langsley, Stefan W. Hell, Vernon B. Carruthers, David J. P. Ferguson, Markus Meissner

**Affiliations:** 1 Institute of Infection, Immunity and Inflammation, Wellcome Centre for Molecular Parasitology, Glasgow Biomedical Research Centre, University of Glasgow, Glasgow, United Kingdom; 2 Department of NanoBiophotonics, Max Planck Institute for Biophysical Chemistry, Göttingen, Germany; 3 German Cancer Research Center/BioQuant, Heidelberg, Germany; 4 Department of Microbiology and Immunology, University of Michigan School of Medicine, Ann Arbor, Michigan, United States of America; 5 Parasitology, Department of Infectious Diseases, University of Heidelberg Medical School, Heidelberg, Germany; 6 Walter and Eliza Hall Institute of Medical Research, Melbourne, Victoria, Australia; 7 Laboratoire de Biologie Cellulaire Comparative des Apicomplexes, Institut Cochin, Inserm, U567, CNRS, UMR 8104, Faculté de Médecine Paris V – Hôpital Cochin, Paris, France; 8 Nuffield Department of Pathology, University of Oxford, John Radcliffe Hospital, Oxford, United Kingdom; Univeristy of California, Los Angeles, United States of America

## Abstract

The basic organisation of the endomembrane system is conserved in all eukaryotes and comparative genome analyses provides compelling evidence that the endomembrane system of the last common eukaryotic ancestor (LCEA) is complex with many genes required for regulated traffic being present. Although apicomplexan parasites, causative agents of severe human and animal diseases, appear to have only a basic set of trafficking factors such as Rab-GTPases, they evolved unique secretory organelles (micronemes, rhoptries and dense granules) that are sequentially secreted during invasion of the host cell. In order to define the secretory pathway of apicomplexans, we performed an overexpression screen of Rabs in *Toxoplasma gondii* and identified Rab5A and Rab5C as important regulators of traffic to micronemes and rhoptries. Intriguingly, we found that not all microneme proteins traffic depends on functional Rab5A and Rab5C, indicating the existence of redundant microneme targeting pathways. Using two-colour super-resolution stimulated emission depletion (STED) we verified distinct localisations of independent microneme proteins and demonstrate that micronemal organelles are organised in distinct subsets or subcompartments. Our results suggest that apicomplexan parasites modify classical regulators of the endocytic system to carryout essential parasite-specific roles in the biogenesis of their unique secretory organelles.

## Author Summary

Eukaryotic cells evolved a highly complex endomembrane system, consisting of secretory and endocytic organelles. In the case of apicomplexan parasites unique secretory organelles have evolved that are essential for the invasion of the host cell. Surprisingly these protozoans show a paucity of trafficking factors, such as Rabs and it appears that they lost several factors involved in endocytosis. Here, we demonstrate that Rab5A and Rab5C, normally involved in endocytic uptake, actually regulate secretion in *Toxoplasma gondii*, since functional ablation of Rab5A or Rab5C results in aberrant transport of proteins to specialised secretory organelles called micronemes and rhoptries. Furthermore, we demonstrate that independent transport routes to micronemes exist indicating that apicomplexans have remodelled Rab5-mediated vesicular traffic into a secretory system that is essential for host cell invasion.

## Introduction

Eukaryotic cells evolved a complex internal membrane system, giving rise to specialised organelles that are linked to the endocytic or exocytic pathway. The basic organisation of the endomembrane system is conserved in all eukaryotes and includes the ER, Golgi and major exocytic pathways [1]. Indeed, recent efforts to compare trafficking factors in the genome of diverse taxa provided compelling evidence that the endomembrane system of the last common eukaryotic ancestor (LCEA) was very complex with most trafficking factors, like Rab-GTPases, present [2], [3].

Rabs constitute the largest family of small G-proteins that function as molecular switches in vesicular traffic [4]. They are required for the specific transport of vesicles from a donor to an acceptor compartment. Importantly, several studies suggest that the function of orthologous Rabs is highly conserved across different taxa. For example, Rab1 and Rab2 appear to have a highly conserved role in ER/Golgi transport [5], [6] and are present in most eukaryotes. Similarly, at least one copy of Rab5, Rab6, Rab7 and Rab11 can be found across most taxa [2]. Other Rabs are present in different taxa, but have been secondarily lost in some species. A good example is Rab4 that although present in many diverse eukaryotes has been lost in several instances [2]. Others like Rab5 or Rab11 display lineage specific expansion most likely due to gene duplications [7], [8]. This expansion of trafficking factors allowed an increase in organellar complexity in the respective species [9] consistently, in more complex organisms the number of Rabs increased [10]. However, this view can be challenged by the fact that many unicellular organisms contain a huge repertoire of Rabs. A recent comprehensive analysis of 56 Rabs in the ciliate *Tetrahymena thermophila* suggests that this protozoan evolved a highly dynamic flexibility in vesicular trafficking pathways [3], [11]. Similarly, the ciliate *Paramecium tetrauelia* contains more than 200 Rabs [12].

Ciliates and apicomplexans belong to the recently recognised infrakingdom alveolata [13]. Despite the huge expansion of Rab-GTPases seen in ciliates, apicomplexan parasites show only a relatively basic set of Rabs [14]. Only few members, like Rab1A or Rab11B have been described as unique to apicomplexans [8], [15]. Furthermore, it appears that apicomplexans lost other components of their vesicular trafficking system, such as adaptin complex genes [16], or components of the Endomembrane Sorting Complex (ESCRT) [17], [18]. The ESCRT-system consists of four distinct complexes (ESCRT0, -I, -II and -III) and is required for ubiquitin-mediated endocytosis of surface proteins [19]. Although this paucity might suggest the absence of a functional endocytic system, endosomal like compartments [20] and lysosome-like vacuoles have been described [21], [22]. We recently demonstrated the role of the dynamin related protein B (DrpB) in vesicular traffic to the rhoptries and micronemes and showed that in its absence, organelle-specific proteins enter the constitutive secretion pathway [23]. Similarly, a recent study identified the *T.gondii* homologue of yeast VPS10 (TgSORTLR) as an essential cargo receptor to transport microneme and rhoptry proteins from the Golgi to endosomal-related compartments [24]. As is with DrpB, ablation of VPS10 resulted in the constitutive secretion of microneme and rhoptry proteins and an organellar biogenesis defect. While these studies confirm the crucial role of the Golgi/TGN (trans-Golgi network) and the highly conserved retromer complex in the specific transport of microneme and rhoptry proteins *en route* to the endosomal-like compartments (ELCs), it is unknown how the parasite coordinates the transport to and from the ELCs.

Given their crucial role as regulators of vesicular transport we performed a systematic analysis of *T.gondii* Rabs to examine their role in the delivery of proteins to the specialised secretory organelles. We found that overexpression of either wild type and/or dominant negative expression of Rab5A and RabC mutants resulted in a specific defect in transport to micronemes and rhoptries. Surprisingly, only a subset of microneme specific proteins requires Rab5A and Rab5C for their transport to the apical complex. Together our data suggest that apicomplexans have altered part of their endocytic system into unique secretory organelles.

## Results

### 12 Rabs are expressed in the tachyzoite stage

Previously, 15 genes encoding Rab-like proteins have been identified in the genome of *T. gondii*, whereas analyses of other apicomplexan genomes indicated the presence of 9 Rab-GTPases in *Theileria*, *Cryptosporidium* and *Babesia* and 11 in *Plasmodium* [14] (Table S1). We excluded 3 of the previous identified putative Rab-GTPases in *T.gondii* from our analysis based on the absence of Rab-family specific motifs or expression evidence in the available database (www.toxoDB.org) and our inability to amplify cDNA for the respective genes (Table S2). We amplified full-length cDNA for each *Tgrab* and verified their predicted amino acid sequence (Figure S1, Table S1&S2).

### Phylogenetic analysis of apicomplexan Rabs

**Figure 1:**
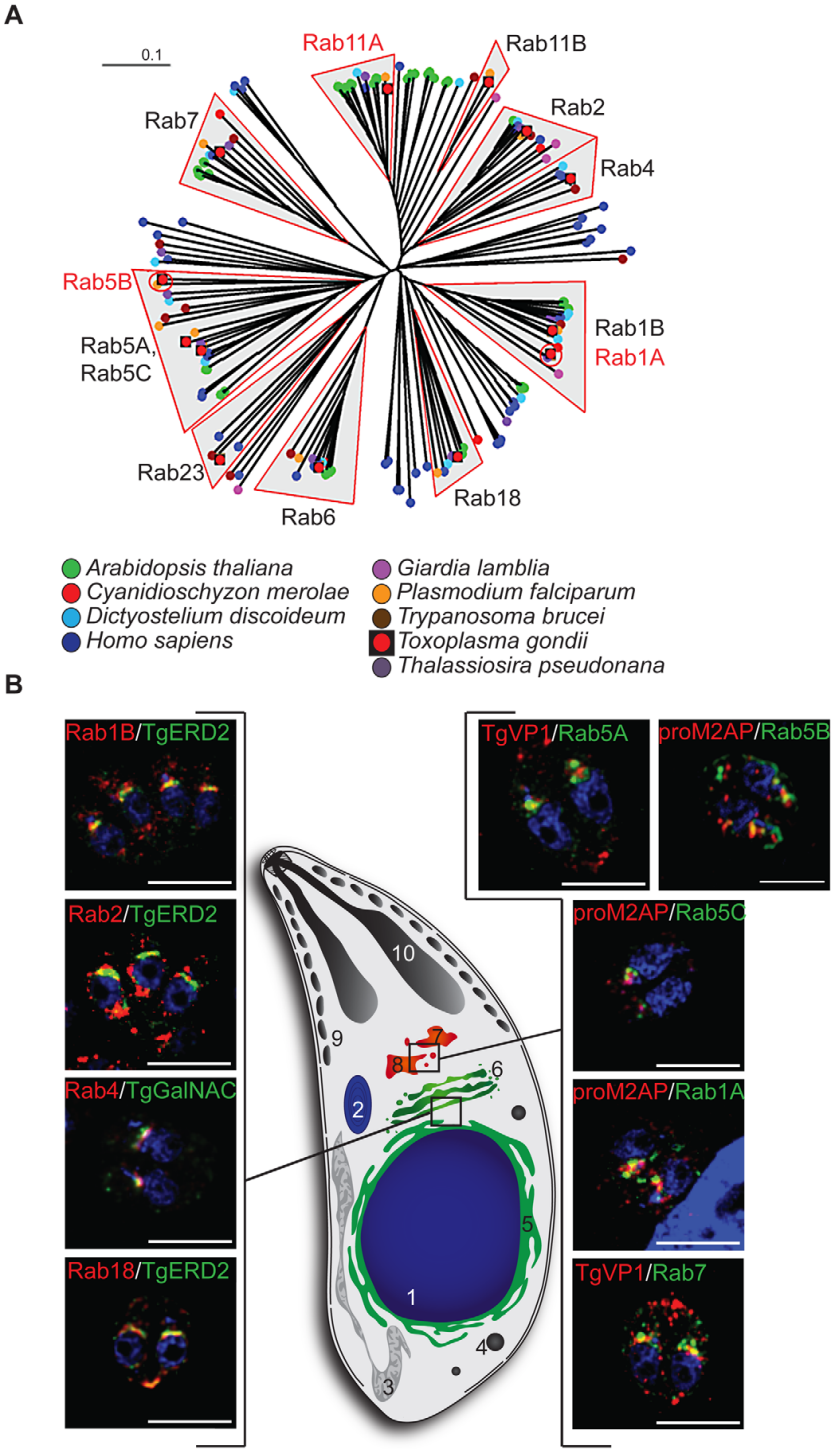
Only a basic set of Rab-GTPases is present in apicomplexan parasites. (A) Unrooted neighbour joining tree of the GTPase domains identified from each conserved Rab sequence of the eight canonical species and *T.gondii*. Clades containing sequences derived from *T. gondii* are indicated by shaded wedges. Three major clades containing *T. gondii* sequences are evident containing sequences of a) Rab1 and18 b) Rab5,6,7 and 23 c) Rab2,4 and 11. The species origin of the nodes is shown by a colour code as indicated. For detailed subtrees of major clades and support values see Figure S2). (B) Immunofluorescence analysis of intracellular parasites expressing ddFKBPmyc-Rab1A,B,2,4,5A,5C,7 and 18 or Rab5B-ddFKBPHA-construct treated for 18 hrs with 1 µM Shld-1 prior to fixation. The left panel shows Rabs costained with ER/Golgi marker (TgERD2 and TgGalNAC in green), whereas the right panel shows co-staining with markers for ELCs (TgVP1 and proM2AP in red), the nucleus is shown in blue (Dapi). The scale bars represent 5 µm. 1 =  Nucleus, 2 =  Apicoplast, 3 =  Mitochondrium, 4 =  Dense Granule, 5 =  Endoplasmatic Reticulum (ER), 6 =  Golgi, 7 =  late endosome-like compartment (TgVP1 compartment), 8 =  early endosome like compartment (proM2AP), 9 =  Micronemes, 10 =  Rhoptries. See also Figure S4&S5 for more details.

Given the presence of unique secretory organelles in apicomplexan parasites, we wondered if some of the identified Rabs show unique features that would classify them as apicomplexan or alveolate specific, as previously demonstrated for Rab11B [8], or Rab1A [15]. Surprisingly, it appeared that apicomplexans have a highly conserved, minimal set of Rabs that has been maintained across most eukaryotic lineages (Figure 1A, Figure S2). Importantly, the lack of lineage specific expansions of Rabs in apicomplexans raised the question as to how these parasites have evolved an elaborate endomembrane system with several unique secretory organelles [7], [9], [10]. We speculated that some of the highly conserved Rabs (i.e. Rab4, Rab5, Rab7) might have acquired a novel role in regulating vesicular transport in apicomplexan parasites.

### *T.gondii* Rab-GTPases localise to the early and late secretory system

For the generation of stable transfected parasites expressing Rabs we employed the ddFKBP-system that allows tuneable regulation of protein levels in dependence of the ligand Shield-1 (Shld-1) [25] and hence minimises the risk of mis-localisations due to overexpression of the respective Rab. As *Toxoplasma* Rab6 [26], Rab11A and Rab11B [8], [27] have been described previously they were excluded from this analysis.

We generated parasite lines expressing ddFKBPmyc-tagged versions of Rab1A, 1B, 2, 4, 5A, 5C, 7, 18 and Rab5B-ddFKBPHA and performed co-localisation studies. Hereafter, these transgenic strains are referred to as simply Rab1A, Rab1B, Rab2, Rab4, Rab5A, Rab5B, Rab5C, Rab7 and Rab18. For all experiments we confirmed Shield-1 dependent regulation of protein levels (Figure S3) and adjusted the Shield-1 concentration to a low level to minimise the risk of artefacts due to overexpression of the respective protein. A summary of the different localisations is shown in Figure 1B. Intriguingly, we found that none of the Rabs co-localised with the apical secretory organelles. Instead, we found that all Rabs localise to the early secretory pathway (Rab1B, 2 and 18), the Golgi (Rab4), or the late secretory pathway (Rab5A, Rab5B, Rab5C and Rab7). For Rab1A we were unable to determine its exact localisation, since co-staining with diverse markers of both the early and late secretory system was observed, possibly indicating multiple roles of this Rab (Figure S4). For a more detailed discussion of the individual localisations, see supplements (Text S1 and Figure S4&S5).

### Overexpression of Rab2,4,5A,5B and 5C is deleterious for the parasite

**Figure 2:**
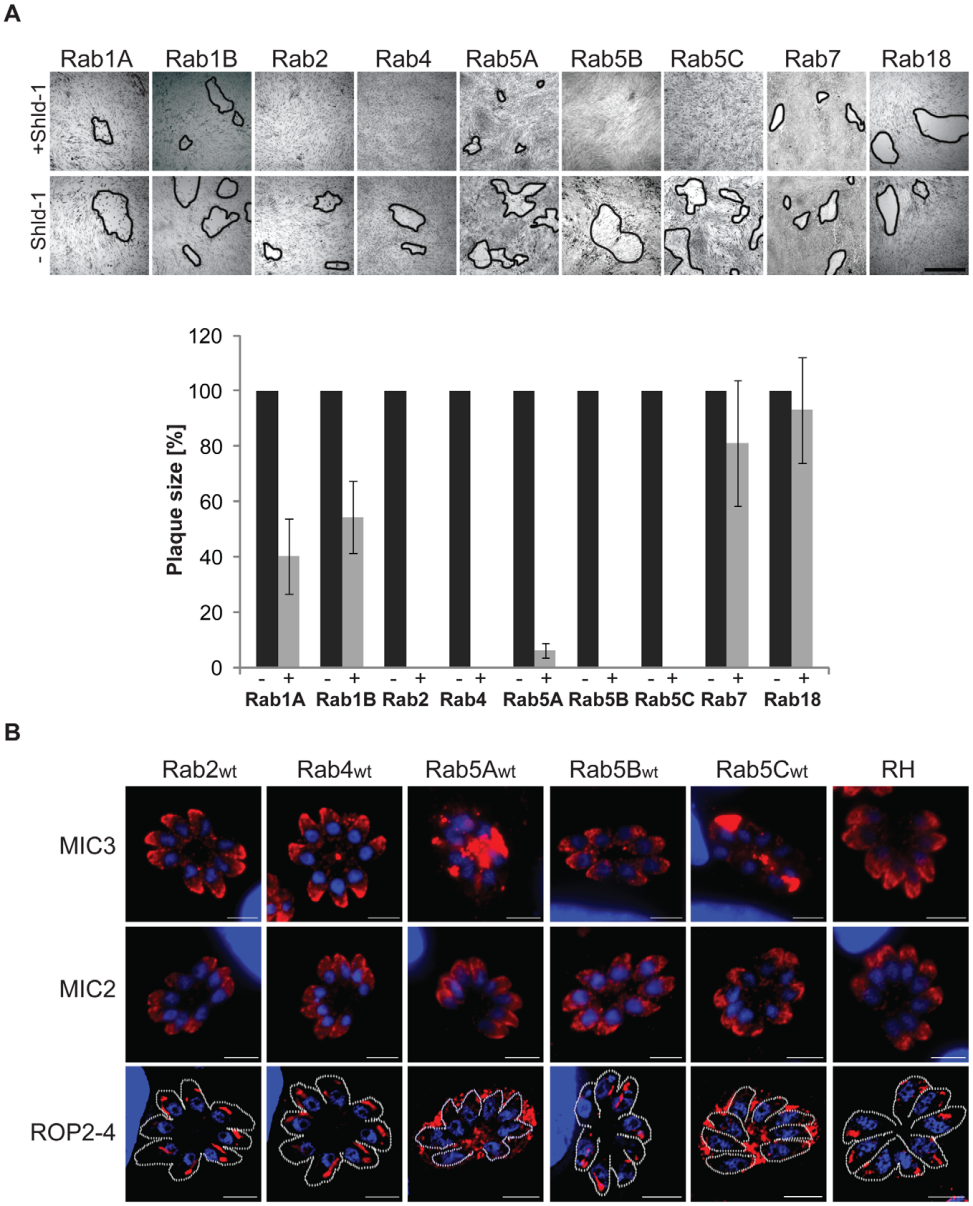
Overexpression of ddFKBPmyc-Rab5A and ddFKBPmyc-Rab5C causes mislocalisation of only a subset of microneme proteins. (A) Growth analysis of parasites expressing indicated ddFKBPmyc-Rab constructs inoculated on HFF cells and cultured for 5 days +/− 1 µM Shld-1. Single plaques are indicated by black edging. The scale bar represents 1 mm. The depicted quantification of the plaque sizes is a representative of 3 independent experiments. In each case, the mean area and standard deviation of 10 plaques was determined. The data was normalised relative to the plaque size of the respective uninduced parasite strain. (B) Immunofluorescence analysis of intracellular parasites expressing indicated ddFKBPmyc-Rab constructs and wild type parasites RH^hxgprt−^treated for 24 hrs with 1 µM Shld-1 and probed with α-MIC3, α-MIC2 or α-ROP2-4 antibody (red) and Dapi (blue). Only parasites overexpressing Rab5A and 5C show a mislocalisation effect on the secretory organelles (micronemes, rhoptries). Scale bars represent 5 µm.

Overexpression of Rab-GTPases is an efficient method to analyse their function and has been successfully employed in different eukaryotes [28], [29]. To screen for phenotypes caused by overexpression of the different ddFKBPmyc/HA-tagged Rabs, we inoculated each of the above transgenic parasite strains on HFF monolayers for 5 days in presence and absence of 1 µM Shield-1, which ensured maximal stabilisation of the respective ddFKBP-tagged protein [25] (Figure S3) and found that parasite growth was ablated for Rab2,4 5A,5B and 5C (Figure 2A). Rab1A and Rab1B displayed a growth defect, while in contrast for Rab7 and Rab18 only minor differences in parasite growth were detected (Figure 2A)

### Rab5A and Rab5C are required for transport to apicomplexan-specific secretory organelles

In order to identify Rabs that play a crucial role in vesicular transport to the apicomplexan-specific secretory organelles we grew parasites overexpressing Rabs 2,4,5A,5B and 5C for 24 hours in presence of Shield-1 and analysed the location of the microneme proteins MIC2 and MIC3 and of the rhoptry proteins ROP2,3 and 4 (Figure 2B). While we were unable to detect aberrant targeting of these proteins in Rab2, Rab4 and Rab5B parasites, overexpression of Rab5A and 5C resulted in an aberrant localisation of rhoptry proteins and MIC3 in the lumen of the parasitophorous vacuole. This indicates that these proteins have entered the constitutive secretory pathway in these parasites. Curiously, we were unable to detect a similar defect in trafficking of MIC2, which displayed a normal microneme location in all lines (Figure 2B). We conclude therefore that overexpression of Rab5A and Rab5C results in a specific trafficking defect to rhoptries and micronemes and the different behaviour of MIC2 and MIC3 suggest that specific transport pathways exist for a subset of microneme proteins.

### Expression of trans-dominant Rab1A and Rab7 has no influence on secretory organelles

Similar to Rab5A and 5C we found Rab1A and Rab7 on post-Golgi membranes (Figure 1B, Figure S4&S5), indicating a role in the late secretory pathway. We established parasites conditionally expressing ddFKBPmyc-tagged, trans-dominant versions of Rab1A and Rab7 (Figure S6&7). Expression of dominant negative Rab1A(N126I) resulted only in a slight, but not significant growth defect (Figure S6) and no adverse effects were evident on secretory or other organelles (data not shown).

The high conservation in eukaryotes and its localisation to ELCs in apicomplexans suggests an important role for Rab7, possibly during the vesicular transport to a plant like vacuole and/or secretory organelles [21], [22]. We generated parasite strains conditionally expressing dominant negative (N124I) and dominant active (G18E) [30] Rab7 (Figure S7). While expression of Rab7(N124I) did not affect parasite proliferation, expression of Rab7(G18E) blocked growth (Figure S7). However, this growth deficiency was not apparently linked to vesicle targeting to secretory organelles, since all markers tested (proM2AP, TgVP1, TgCPL, M2AP, MIC3, Rop2-4) showed a normal localisation in parasites expressing Rab7(G18E) (Figure S7). Therefore, in the absence of additional markers for the ELCs we were unable to define the precise trafficking step regulated by Rab7 during the asexual life cycle of the parasite.

### Expression of dominant negative Rab5B causes no mislocalisation of micronemal and rhoptry proteins

Although Rab5B showed a similar localisation at the ELCs as Rab5A and Rab5C, we failed to detect an immediate effect on trafficking to the micronemes or rhoptries (Figure 2B). However, since prolonged overexpression of Rab5B-ddFKBPHA resulted in the gradual mislocalisation of microneme and rhoptry proteins (Figure S8), we wished to exclude a direct role of Rab5B in the transport to the secretory organelles. Therefore, we generated transgenic parasites conditionally expressing a dominant negative version of Rab5B (ddFKBPmyc-Rab5B(N152I)). As expected, we found that parasite growth was almost completely blocked (Figure S8). However, no effect on the location of microneme and rhoptry proteins could be detected, demonstrating that Rab5B plays no direct role in the vesicular transport to the secretory organelles of the parasite (Figure S8).

### Overexpression of Rab5A and Rab5C results in the mislocalisation of only a subset of microneme proteins

Since we found in the initial screen that overexpression of both Rab5A and Rab5C resulted in the constitutive secretion of MIC3, but not MIC2 (Figure 2B), we analysed further the localisation of different microneme proteins to determine their dependence on functional Rab5A and Rab5C. While MIC2, M2AP and AMA1 showed normal localisation in the micronemes, MIC3, 8 and 11 entered the constitutive secretory pathway upon overexpression of Rab5A and Rab5C (Figure S9). In good agreement with other mutants that show a defect in microneme and rhoptry biogenesis [23], [24], we found that parasites overexpressing Rab5A or Rab5C displayed no proliferation defect, while host cell egress and invasion was significantly blocked (Figure S9).

### Expression of dominant negative Rab5A and Rab5C

**Figure 3:**
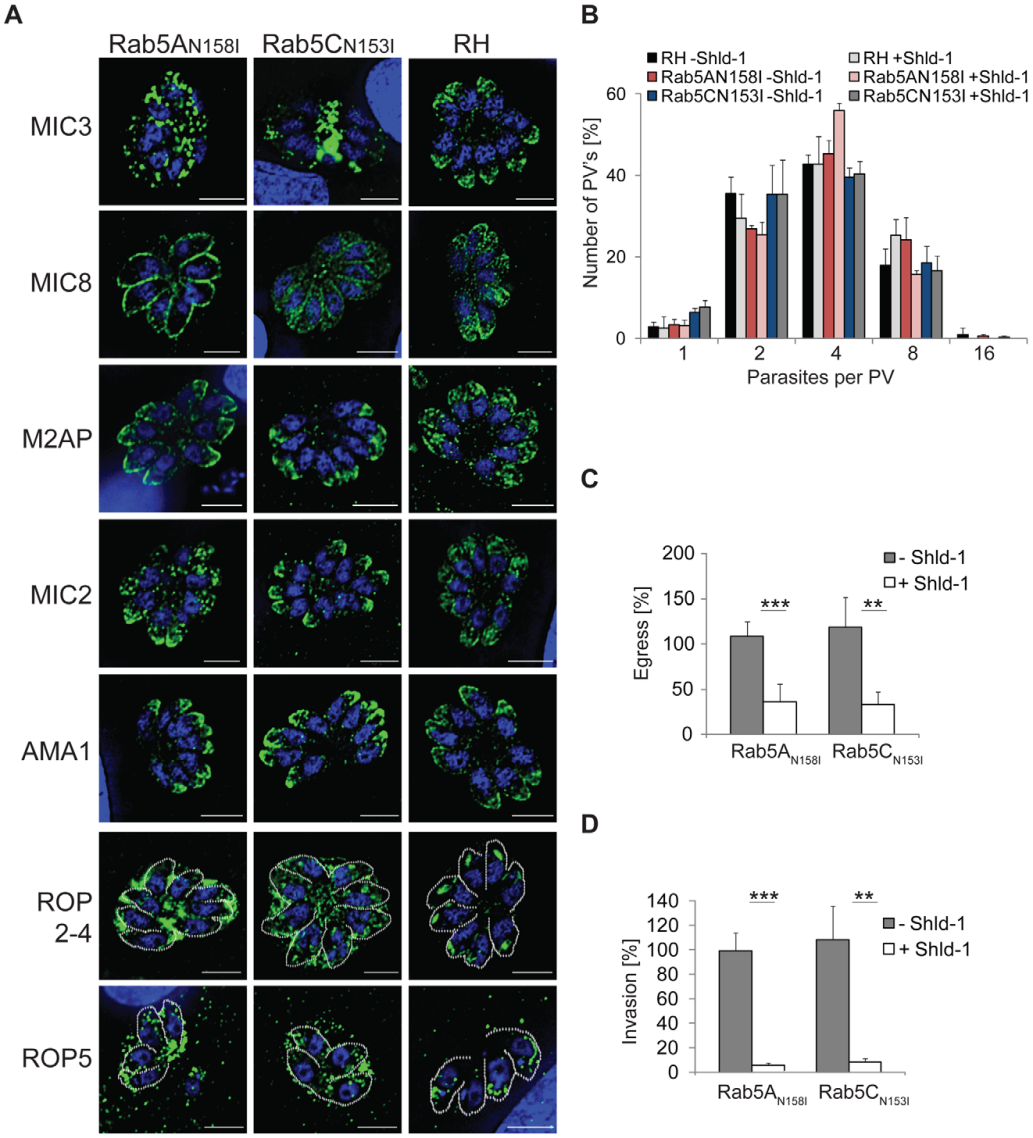
Analysis of parasites expressing ddFKBPmyc-Rab5A(N158I) and ddFKBPmyc-Rab5C(N153I). (A) Immunofluorescence analysis of indicated parasites treated for 24 hrs with 1 µM Shld-1 and probed with indicated antibodies (green) and Dapi (blue). (B) Replication assay of indicated parasites grown for 24 hrs in presence, or absence of 1 µM Shld-1 prior to fixation. Average number of parasites per PV was determined. No significant differences in replication were detected. (C) Egress assay of indicated parasites grown for 36 hrs +/− 1 µM Shld-1 before egress was triggered with A23187. Host cell lysis was determined 8 min after induction of egress and normalised with RH ^hxgprt−^parasites. For both mutants the egress is significantly decreased. (D) Invasion assay of indicated parasites treated for 24 h +/− 1 µM Shld-1, scratched and inoculated on fresh HFF cells. Subsequently invasion was determined and normalised with RH^hxgprt−^ parasites. For both mutants the invasion is significantly blocked. (B–D) Mean values and the respective standard deviation of three independent experiments are presented. (***indicates p-value of P≤0.01 and **indicates P≤0.02 in a two tailed Student's test).

To ensure specificity of the observed overexpression phenotype, we established parasites expressing dominant negative versions of Rab5A(N158I) and Rab5C(N153I). We verified efficient Shield-1 dependent regulation of both dominant negative Rab mutants and found a severe block in parasite growth upon their induction (Figure S10). Expression of dominant negative Rab5A(N158I) and Rab5C(N153I) resulted in a phenotype identical to that observed for overexpression of their wild type versions. We found that all rhoptry proteins analysed (Rop2-4,5) enter the constitutive secretory pathway. In sharp contrast, only a subset of micronemal proteins (MIC3,8,11) were mislocalised upon expression of Rab5A(N158I) and Rab5C(N153I) (compare Figure 3 and Figure S9). While intracellular replication was not affected (Figure 3B), we observed that the mislocalisation of essential microneme and rhoptry proteins results in parasites that are blocked in host cell egress (Figure 3C) and invasion (Figure 3D). This reconfirms that secretory organelles function primarily during host cell egress and invasion and are at least *in vitro* dispensable for parasite replication [23], [24].

**Figure 4:**
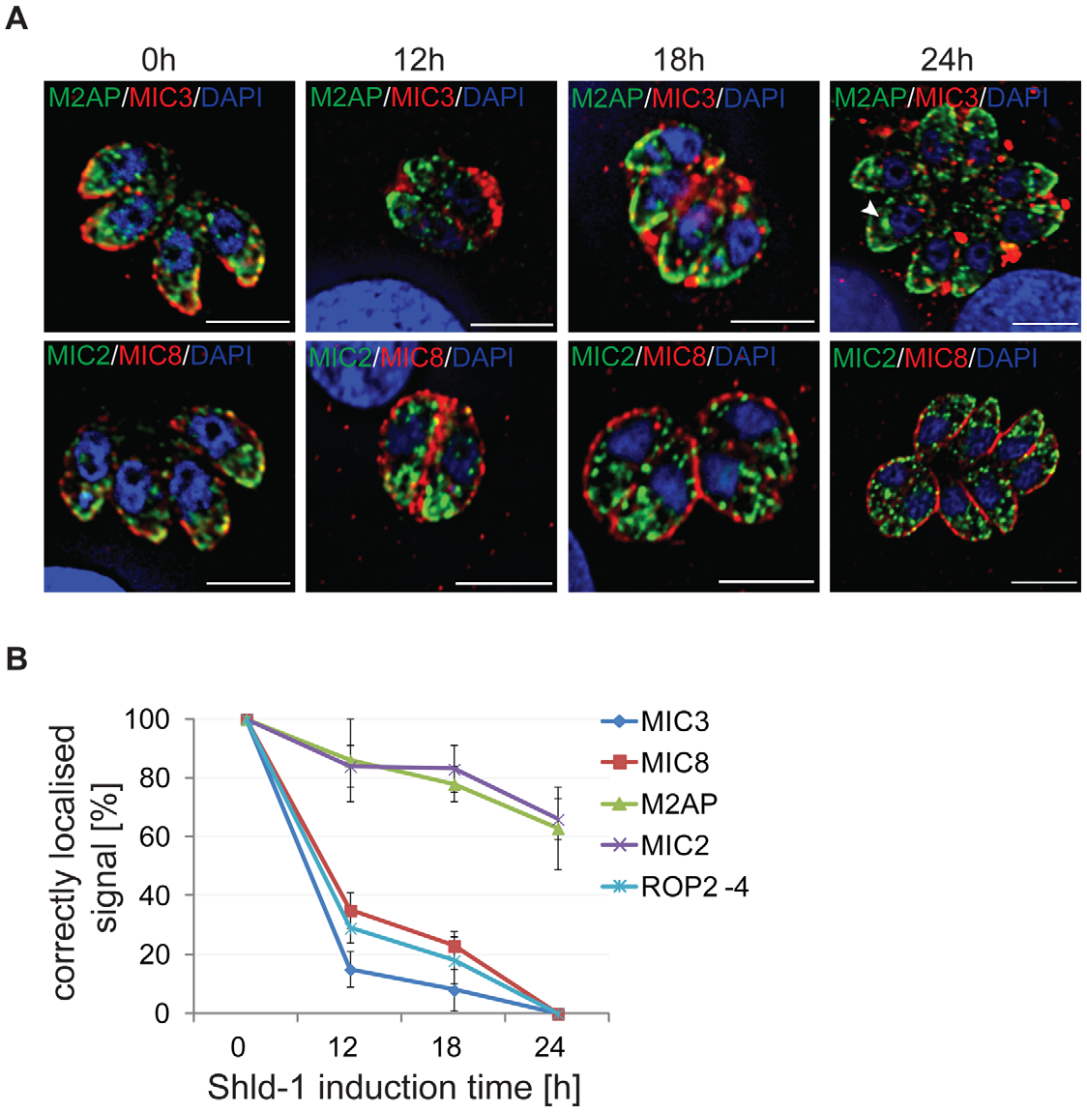
Time course analysis of parasites expressing ddFKBPmyc-Rab5A(N158I). (A) Immunofluorescence analysis of intracellular parasites stably expressing ddFKBPmyc-Rab5A(N158I) treated for 0, 12, 18 and 24 hrs with 1 µM Shld-1 and co-stained with the indicated microneme antibodies (green/red) and Dapi (blue). (B) Quantification of localisations of Rop2-4 and indicated microneme proteins. 300–400 PVs of three independent experiments were analysed and normalised with RH ^hxgprt−^parasites. Mean values and the respective standard deviation are presented. Note that MIC2 and M2AP is mainly mislocalised inside the parasite (arrowhead in A).

Next, we performed a time course analysis following Shield-1 induction and found MIC3 and MIC8 to be aberrantly targeted within 12 hours (∼80%), whereas ∼20% of parasites showed mislocalisation of MIC2 and M2AP after this time (Figure 4A and B). Importantly, in these cases we found that MIC2 and M2AP show a non-specific, intracellular localisation (Figure 4A, arrowhead), whereas MIC3 and MIC8 are trafficked via the constitutive secretory pathway (Figure 4A and B).

We also verified that other organelles, such as Golgi, apicoplast, mitochondria, dense granules and the inner membrane complex (IMC) are not affected by expression of dominant negative Rab5A (Figure S11) or Rab5C (data not shown), demonstrating that Rab5A and Rab5C are specifically required for the transport of rhoptry proteins and a subset of microneme proteins.

### Rab5A and Rab5C are not required for microneme processing and organisation of endosomal-like compartments in *T.gondii*

**Figure 5:**
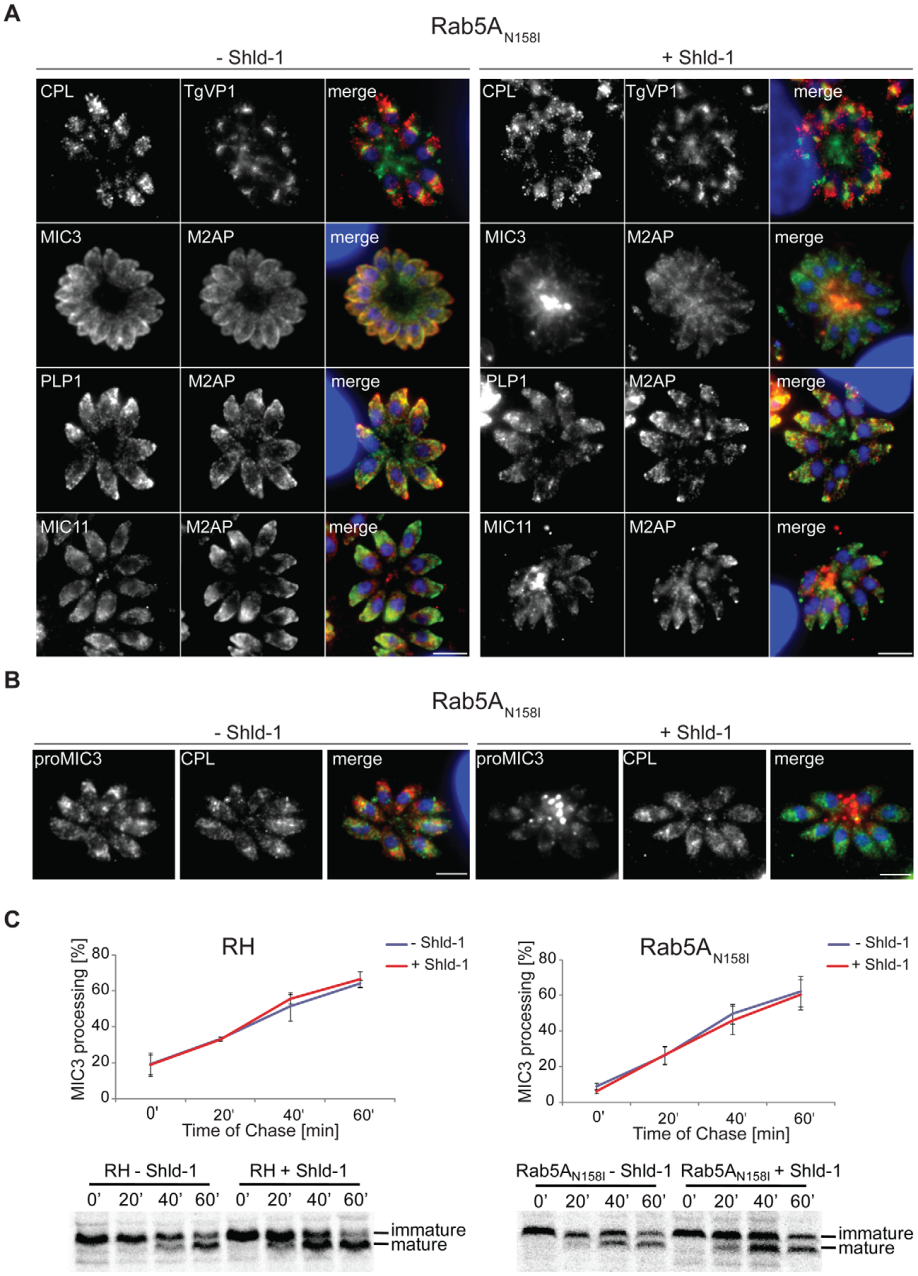
Microneme processing and organisation of endosomal-like compartments (ELCs) is unaffected in ddFKBPmyc-Rab5A(N158I) expressing parasites. (A) Parasites expressing ddFKBPmyc-Rab5A(N158I) have been grown for 24 hrs +/− 1 µM Shld-1 and analysed with the indicated antibodies. MIC3 and MIC11 are not transported to the micronemes, whereas the micronemal proteins PLP1 and M2AP as well as the marker for endosomal-like compartments (CPL and TgVP1) show normal localisation. The scale bars represent 5 µm. (B,C) Pulse-chase experiments of MIC3 processing in parasites expressing ddFKBPmyc-Rab5A(N158I). (B) Immunofluorescence analysis of ddFKBPmyc-Rab5A(N158I) parasites grown for 18 hrs +/− 1 µM Shld-1 and probed with proMIC3 (red) and CPL (green) antibodies. In presence of the inducer the pro-peptide of MIC3 is secreted into the PV (proMIC3), whereas no effect on endosomal-like compartments (CPL) is obvious. Dapi is shown in blue. The scale bar represents 5 µm. (C) Quantification and the respective western blots of MIC3 maturation in RH ^hxgprt−^ and ddFKBPmyc-Rab5A(N158I) parasites are shown. Mean values and the respective standard deviation of three independent experiments are presented.

To gain more insight into the role of Rab5A and RabC in vesicular transport, we analysed the localisation of ELC markers, such as proM2AP, TgCPL and TgVP1 [21], [22]. None of these markers showed a significant relocation when either wild type or dominant-negative Rab5A and Rab5C were expressed (Figure 5A), indicating that in *T.gondii* functional ELCs are maintained in the absence of functional Rab5A or Rab5C.

Many microneme proteins, such as MIC3 and M2AP undergo proteolytic maturation during their transit through the ELCs [20], [22], [31]. Since MIC3, but not M2AP is constitutively secreted in parasites expressing dominant negative Rab5A, we investigated at which step this rerouting occurs. If rerouting occured directly at the Golgi MIC3 would be secreted as an immature proMIC3. In contrast, if rerouting occurred at the ELCs, processing of the propeptide would take place, resulting in secretion of mature MIC3. Therefore, we performed a pulse-chase experiment and compared pro-peptide processing of MIC3 in wild type (RH) parasites and parasites expressing ddFKBPmyc-Rab5A(N158I) in presence and absence of Shield-1 (Figure 5B). We were unable to detect any differences in propeptide processing, strongly suggesting that the rerouting of MIC3 occurs post-Golgi, after processing in the ELCs (Figure 5C).

### Microneme proteins are transported in different types of vesicles

**Figure 6:**
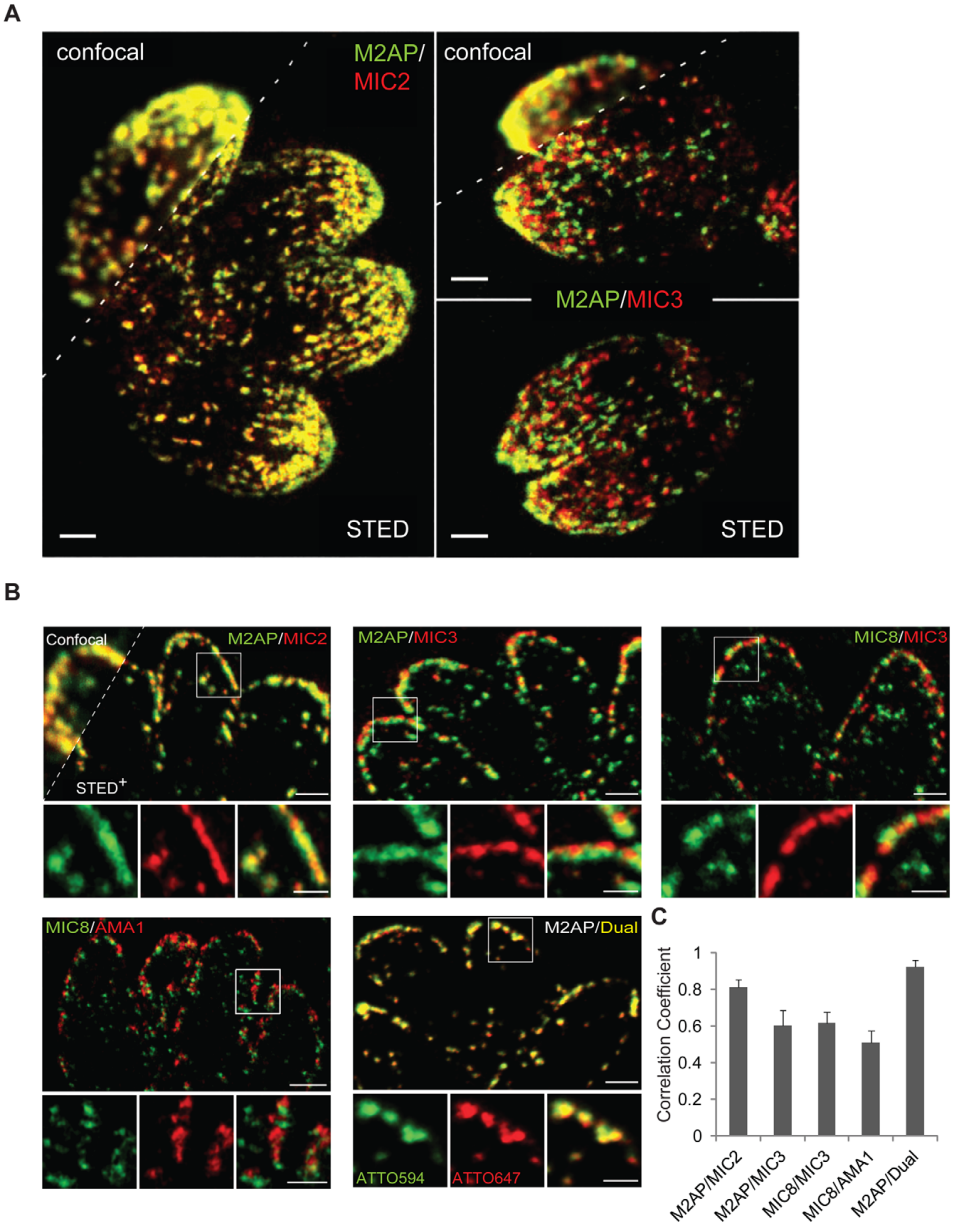
Two-colour STED analysis reveals different localisiations of microneme proteins in wild type (RH^hxgprt−^) parasites. (A) Two-colour STED (Stimulated Emission Depletion) measurements on intracellular wild type (RH ^hxgprt−^) parasites reveals high-resolution of microneme organelles at the apical complex. Resolution obtained in confocal mode does not allow discerning single organelles at the densely packed apical pole of *T.gondii* while STED microscopy enables a precise (co-)localisation analysis. Dual labelling of MIC2 and M2AP, and M2AP and MIC3 on whole-mount parasites shows a perfect co-localisation for the complex forming microneme proteins MIC2 and M2AP, but M2AP and MIC3 exhibit distinct localisations. Scale bars represent 1 µm. (B) Two-colour STED images of typical microneme co-localisation patterns in 100 nm ultrathin sections. Intracellular wild type (RH ^hxgprt−^) parasites were immunolabelled with indicated antibody combinations. Confocal and STED^+^ (linear deconvoluted) resolution is shown for M2AP/MIC2). As a positive co-localisation control dual labelling of M2AP with a mixture of ATTO 594-and ATTO 647N-labelled secondary antibodies (M2AP/Dual) was performed. Scale bars for the overview images represent 1 µm and for close-up images 0.5 µm. (C) Quantification of the correlation between indicated micronemal marker proteins from (B) by calculating the Pearson's correlation coefficient. M2AP-Dual represents the positive control for the correlation analysis.

The results suggest that microneme proteins reach their destination using at least two distinct transport routes, with one depending on functional Rab5A and/or Rab5C. Consequently we speculated that micronemes might be organised into different subsets with different protein content. Dense clustering of secretory organelles within the apical complex of the parasite and limitations in optical resolution make it difficult to differentiate individual compartments using standard microscopy techniques. For these reasons we used super-resolution two-colour STED (Stimulated Emission Depletion) microscopy [32] to finely pinpoint the subcellular localisation of microneme proteins (Figure 6). As expected MIC2 and its interaction partner M2AP [33] exhibit an extensive co-localisation pattern (Figure 6A). In contrast, M2AP and MIC3 localised to independent subsets with minimal overlap in the sub-apical region of the parasites (Figure 6A). This is in good agreement with the genetic data presented above and demonstrates that MIC3- and M2AP-positive vesicles are independently transported to the apical tip of the parasite. Due to limitations in z-resolution, we were unable to resolve if MIC3 and M2AP show also different locations at the densely packed apical tip of the parasite. Therefore we performed two-colour STED on 100 nm ultrathin-melamine sections of parasites labelled with antibodies against different microneme proteins (Figure 6B). Examination of thin-sections of the apical tip revealed a near perfect co-localisation of MIC2 and M2AP (as expected), whereas all other microneme protein combinations exhibited lower co-localisation correlations (Figure 6B and C). Together, the results demonstrate that microneme proteins are transported via independent routes to the micronemes, where they are stored either in different subsets or sub-compartments, rather than a unique, homogenous organelle.

### Ultrastructural analysis on parasites expressing Rab5A(N158I)

**Figure 7:**
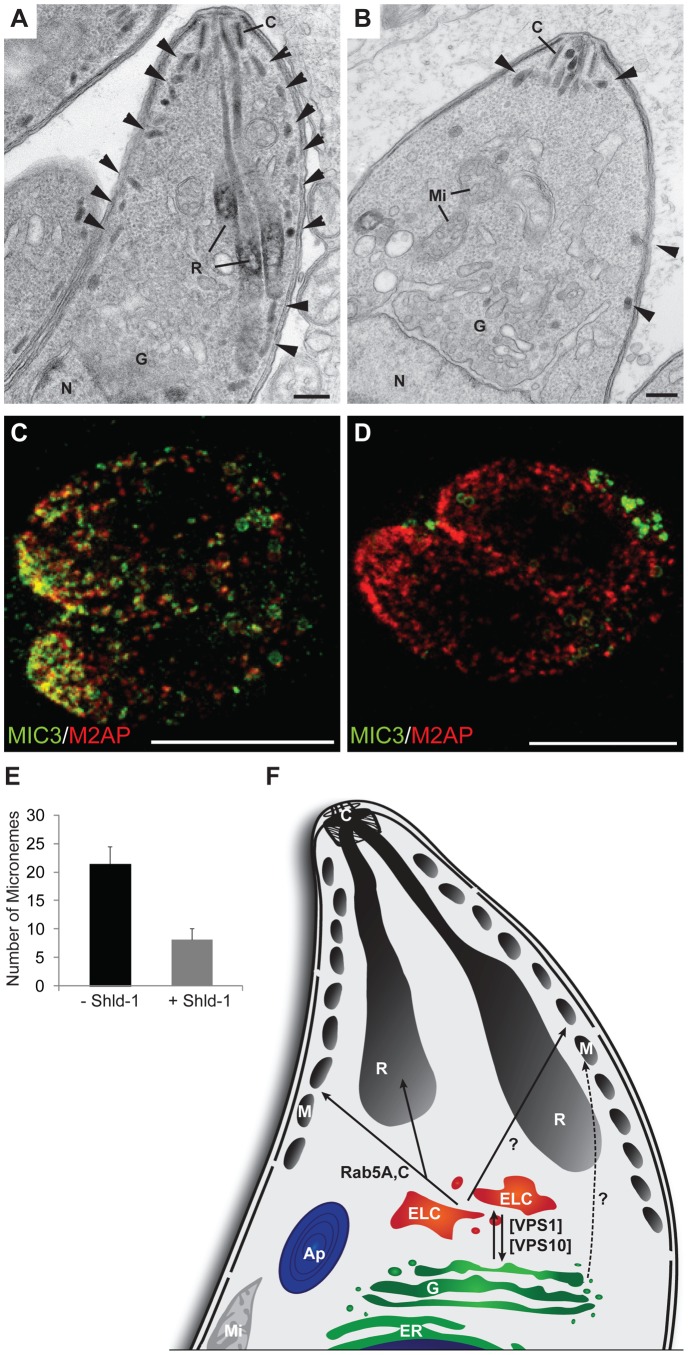
Ultrastructural and STED analysis of parasites expressing ddFKBPmyc-Rab5A(N158I). (A) Electron microscopy analysis of the apical region of a tachyzoite expressing ddFKBPmyc-Rab5A(N158I) in the absence of 1 µM Shld-1 showing the natural morphological organisation of the apical organelles with various number of micronemes (arrowhead) associated with the limiting membrane complex. (B) Similar parasite stage from a culture incubated for 24 hrs with 1 µM Shld-1 showing a reduced number of micronemes (arrowheads). Scale bars represent 100 nm. (C,D) STED analysis of the same parasite strain grown in absence (C) or presence (D) of 1 µM Shld-1 for 18 hrs. Two-colour STED measurements were performed on MIC3 and M2AP, showing the absence of MIC3 within the parasites expressing ddFKBPmyc-Rab5A(N158I). (E) Electron microscopy quantification of micronemes present in longitudinal sections passing through the conoid and nucleus. 20 parasites per situation were quantified. Less micronemes are detected when ddFKBPmyc-Rab5A(N158I) is expressed. (F) Model of the vesicular traffic of secretory proteins to the rhoptries and micronemes. After modification at the Golgi secretory proteins are transported to the ELC's, which is regulated by VPS1 and VPS10. From the ELC's some of the microneme proteins (i.e. MIC3, MIC8, MIC11) and rhoptry proteins are transported to their target organelles in a Rab5A/C dependent manner. The regulation of the transport of other microneme proteins (AMA1, MIC2, M2AP, PLP1) remains unknown (?). VPS10 =  Sortilin (TgSORTLR), VPS1 =  dynamin related protein B (DrpB), Ap =  Apicoplast, C =  Conoid, ELC =  endosome like compartments, ER =  Endoplasmatic Reticulum, G =  Golgi, Mi =  Mitochondrium, M =  Micronemes, R =  Rhoptries.

Next, we analysed the ultrastructure of parasites overexpressing Rab5A(N158I). In good agreement with our previous analysis, these parasites are devoid of rhoptries and only very few micronemes are identified (Figure 7A,B). In particular, interference with Rab5A function resulted in a significant loss of micronemes (∼70%) (Figure 7E). STED analysis showed that M2AP has a normal localisation at the apical pole of the parasite, whereas MIC3 is now detected in the parasitophorous vacuole (Figure 7D). Together, these results indicate that M2AP is transported to a subset of micronemes in a Rab5A-independent manner, while MIC3 transport cannot occur in absence of functional Rab5A.

## Discussion

As Apicomplexa parasites invade the host cells, microneme content is the first to be released, as they contain virulence factors that act in a sequential manner during host cell egress and reinvasion. The microneme transmembrane protein MIC2 is involved in host cell attachment and gliding motility [34] and MIC8 is required for rhoptry secretion [35] leading to the establishment of the moving junction (MJ). Finally, AMA-1 has been suggested to interact with the MJ [36], [37]. How *Toxoplasma* achieves sequential secretion of proteins present in the same organelle has remained a mystery. This is partly due to the dense packing of secretory organelles within the apical complex of the parasite that limits their optical resolution, rendering it difficult to differentiate between individual sub-compartments using standard microscopy. To test whether micronemes are in fact made up of multiple subsets we employed two-colour STED measurements on ultra-thin sections to finely pinpoint the location of different microneme proteins. We found that only few microneme proteins, such as MIC2 and M2AP that are known to form a complex [38], co-localise. In contrast substantially less co-localisation was observed for several other microneme proteins, suggesting the presence of different subsets of micronemes with independent protein content. Alternatively, the differential localisation could reflect organisation of micronemal proteins into sub-compartments, similar to the rhoptries, where proteins are either localised in the bulb, or the neck of the organelle [39].

To identify apicomplexan Rabs involved in regulating vesicular transport to unique parasite secretory organelles, we employed an overexpression screen and identified *T. gondii* Rab5A and Rab5C as key regulators. Intriguingly, we found that Rab5A and Rab5C regulate the localisation of MIC3, 8, 11 and not MIC2, PLP1, AMA1 and M2AP. Combined with our STED analysis it strongly suggests that micronemes are organised into at least 2 different subsets. Similarly, ultrastructural analysis of Rab5A and Rab5C mutants showed only a subset of micronemes to be ablated. In contrast to micronemes, interference with Rab5A and Rab5C function ablated rhoptry organelles indicating that Rab5A and Rab5C are essential for rhoptry biogenesis. Moreover, pulse-chase experiments indicate that the defect in microneme transport occurs post-Golgi, since MIC3 maturation that occurs in the endosomal system [20], [22], [31] was not affected upon functional ablation of Rab5A and Rab5C.

We speculate that rhoptries and one subset of micronemes share the same Rab5A/C trafficking pathway, see model presented in Figure 7F. It appears that apicomplexans altered their endocytic system to evolve unique secretory organelles. Furthermore we demonstrate that microneme proteins consist of at least two independent populations, with distinct transport pathways and subcellular localisations. One involves transport of MIC3, 8 and 11, whereas the other involves MIC2, M2AP, PLP1 and AMA1. It is possible that redundant pathways are in place that can complement functional abrogation of Rab5A and C (Figure 7F).

Unfortunately we were unable to identify the trafficking pathways involved in the transport of the second subset of microneme proteins (MIC2, M2AP, PLP1 and AMA1).

In other eukaryotes, Rab5-GTPases are known to function in the transport of vesicles in the early endocytic pathway [28], where they act as master regulators for endosome biogenesis [40]. In contrast, Rab5A and Rab5C appear to be essential for the transport of proteins to the unique secretory organelles in *T.gondii*, demonstrating a previously unknown functional plasticity that has allowed apicomplexans to evolve micronemes and rhoptries. In good agreement with our data, all microneme and rhoptry trafficking mutants described so far correspond to homologues of the yeast VPS (vacuolar protein sorting) system. The dynamin related protein B (DrpB) is a homologue of VPS1 [23], Sortilin (TgSORTLR) of VPS10 and Rab5A and RabC are homologues of VPS21. In yeast these mutants were identified by screening for transport defects of carboxypeptidase Y (CPY) to the yeast vacuole, which is analogous to the lysosome [41]–[44]. Interestingly, their abrogation in yeast leads to the constitutive secretion of CPY, a phenotype we observed here for rhoptry and microneme proteins. Similarities between rhoptries and secretory lysosomes have been pointed out [45] and it is tempting to speculate that micronemes and rhoptries are derived from lysosomal organelles. Therefore the data presented in this study are consistent with the parasite modifying parts of its endocytic system giving rise to the formation of unique organelles, required for intracellular parasitism.

## Materials and Methods

### Parasite transfection

*T. gondii* tachyzoites (RH *hxgprt* ^−^) were grown in human foreskin fibroblasts (HFF). To generate stable transformants parasites were transfected and selected as previously described [46]. The selections based on pyrimethamine and chloramphenicole resistance were achieved as described previously [47], [48].

### Constructs

For ddFKBPmyc-tagging of Rab-GTPases full-length cDNAs (see Table S3) were introduced into p5RT70DDmycGFP-HXGPRT [25] using indicated restriction enzymes (Table S3). For the generation of trans dominant Rab-GTPases point mutations were introduced using megaprimer method [49] with indicated oligonucleotides (Table S3). For expression of N-terminal tagged TyRab5A the ddFKBPmyc tag in construct ddFKBPmyc-Rab5A was exchanged for the Ty-tag using EcoRI/NsiI.

### Immunofluorescence analysis

HFF monolayers grown on 24 well coverslips were infected with tachyzoites of the strains to be analysed and grown in presence or absence of 1 µM Shield-1 for 12–24 hrs. Immunofluorescence analysis was performed as described [23]. Z-stack images of 0.15 µm increment were collected on a PerkinElmer Ultra-View spinning disc confocal Nikon Ti inverted microscope, using a 100× NA 1.6 oil immersion lens or DeltaVision Core microscope. For deconvolution Huygens or softWoRx software was used. For image processing ImageJ 1.44 and Adobe Photoshop CS4 were utilised. The Pearson's correlation coefficient was calculated using ImageJ or softWoRx software.

### Western blotting

Freshly lysed extracellular parasites were incubated in culture media in presence and absence of 1 µM Shld-1 and incubated for 4–8 hrs. Parasite pellets (corresponding to 2×10^6^ to 5×10^6^ parasites) were analysed with indicated antibodies as described [25].

### Antibodies and fluorescent organellar markers

The following previously described primary antibodies have been used in these analysis: α-IMC1 [50], α- AMA1 [36], Gra9 [51], α-Rop5 [52], α-Rop2,3,4 [53], α-MIC3 [54], α-proMIC3 [31], α-MIC8 [55], α-MIC2 [56], α-M2AP [20], α-MIC11 [57], α-PLP1 [58], α-CPL [22], α-proM2AP [20], α-VP1[21], α-Catalase [59], GRASP-RFP, TgERD2-GFP, GalNac-YFP [60], FNR-RFP [61], HSP60-RFP [61]

### Phenotypic assays

Plaque, Replication, Invasion and Egress assays were performed as previously described [23], [62]

### Pulse-chase analysis of microneme processing

Parasites were grown for 24 hours in presence or absence of inducer. Pulse-chase analysis of MIC3 processing has been performed as previously described [22].

### Electron microscopy

Monolayers were infected with parasites and cultured for 12 and 24 hours in the presence or absence of 1 µM Shld-1 prior to fixation for routine electron microscopy as described previously [23].

### Thin-sectioning

Thin sections of 100 nm were processed as described previously [63]. Briefly, immunolabelled (see below) parasites in HFF monolayers on 12 mm coverslips were embedded in melamine. After polymerization the block with the parasites-containing cells was detached from the glass coverslip and processed with an ultramicrotome (EM UC6, Leica Microsys- tems GmbH, Wetzlar, Germany) into sections of 100 nm thickness.

### Stimulated emission depletion microscopy (STED)

STED immunofluorescence analysis with intracellular parasites was performed using α-mouse ATTO 565 and α-rabbit Dyomics 485 (DY 485 XL) secondary antibodies. STED immunofluorescence analysis of 100 nm thin-sections was performed using the dye pairs ATTO 594 and ATTO 647N (ATTO-TEC, Siegen, Germany) coupled to α-rabbit and/or α-mouse secondary antibodies (Dianova, Hamburg, Germany). The whole-mount samples and thin-sections were embedded in Mowiol 4-88/DABCO mounting media.

### Two-colour STED measurements

The two-colour STED measurements of whole-mount samples were performed on a home-built setup. The two colour channels were realised using the dyes DY 485 XL (excited at 470 nm) and ATTO 565 (excited at 532 nm). Both fluorophores can be efficiently silenced by the same STED wavelength at 647 nm due to the large Stoke's shift of DY 485 XL. Two pulsed laser diodes served as excitation sources (Picoquant, Berlin, Germany) which were triggered by the STED pulses – generated by an actively mode locked (APE, Berlin, Germany) Ar-Kr laser (Spectra Physics-Division of Newport Corporation, Irvine, USA). The synchronized pulses were combined using acousto-optical tunable filters (AOTF) (Crystal Technologies, Palo Alto, USA) and coupled into a microscope (DMI 4000B with an objective lens ACS APO 63x/1.3NA, Leica Microsystems GmbH, Mannheim, Germany) equipped with a three axis piezo stage-scanner (PI, Karlsruhe, Germany) which also imaged the fluorescence signal onto a confocally arranged aperture of a photon counting module (SPCM-AQR-13-FC, PerkinElmer, Canada). The AOTFs also served as fast shutters and independent power controllers for each laser beam as well as a filter system selecting the fluorescence signal. For additional filtering, a band-pass filter (580/40, AHF Analysentechnik, Tübingen, Germany) was used. The doughnut-shaped intensity profile of the STED focus was generated by inserting a glass phase plate (RPC Photonics, NY, USA) which induced a helical phase ramp from 0 to 2 on the initially flat wave front.

Two-colour STED imaging of melamine sections was performed on a home-built setup as previously described [64]. Two excitation lasers at 570 nm +/− 5 nm (for ATTO 594) and 647 nm +/− 5 nm (for ATTO 647N) originated from a single supercontinuum laser source while two additional high-power STED lasers at 711 nm +/− 3 nm and 745 nm +/− 3 nm, respectively, emerged from the same laser source (SC-450-PP-HE, Fianium, Ltd., Southampton, UK). A pulse-interleaved acquisition scheme was used to image both colour channels in a quasi-simultaneous recording mode (25 ns time-shift). The fluorescent signals were detected by two high-sensitive avalanche photodiodes at 620 nm +/− 20 nm (ATTO 594) and 670 nm +/− 15 nm (ATTO 647N). STED images were deconvolved by a linear deconvolution algorithm using the Software ImSpector (www.imspector.de). The Pearson's correlation coefficient (Figure 6C) was calculated using Image J.

### Phylogenetic analysis

Rab-GTPases from apicomplexan parasites have been previously identified [14]. Orthologue groups were obtained from OrthoMCL DB (http://www.orthomcl.org/cgi-bin/OrthoMclWeb.cgi). From each orthologue group sequences were collected if they were derived from the following species groups: **Canonical species** – representative species from the major groups of the eukaryotes (Baldauf) [except for Rhizaria – no genomic data set] *Homo sapiens* (Opisthokonts), *Dictyostelium discoideum* (amoebazoa), *Arabidopsis thaliana* (archaeplastidae,viridiplantae), *Plasmodium falciparum* (alveolates), *Thalassiosira pseudonana* (stamenopiles), *Trypanosoma brucei* (discicristates), *Giardia lamblia* (excavates), Species drawn from the Alveolate group: *Plasmodium bergei, Cryptosproridium parvum, Neospora caninum, Theileria anuulata, Toxoplasma gondii*, Red algae (*C. merolae*). Candidate sequences were assessed for the presence of ras domains using the hmmsearch option of the HMMER package using the profile PF00071 from Pfam. Sequences were checked for the presence of potential prenylation or myristilation sites in the C-terminal region. The ras domains from the conformant sequences were identified by hmmalign option of the HMMER package and extracted as described previously [65]. Blastp searches of the conformant sequences were performed agains the appropriate Uniprot KB species specific proteome sets. Multiple sequence alignment of the set of ras domains was performed by three independent methods; ClustalW [66], t-coffee [67] and hmmalign [68] guided by the appropriate profile. The alignments used the default settings for each method. Alignments were combined under t-coffee, and quality of alignment assessed - columns displaying low consistency (score < 5) or significant numbers of gaps (> 15%) were removed. The phylogeny was visualised as an unrooted neighbour joining tree by the Splits-Tree program. Major clades containing T.gondii sequences were identified, and the alignments of sequences within these clades extracted. The phylogenies were visualised as rooted Neighbour Joining phylograms, using an appropriate *C. merolae* sequences as outgroup. The robustness of the phylogeny was established by bootstrap analysis (1000 iterations).

## Supporting Information

Figure S1 **Alignment of Rab-like proteins of** ***T. gondii*** **.** Rab consensus motifs are shaded in grey. Highly conserved regions are indicated in red (80% similarity) and grey (50% similarity). Putative motifs for C-terminal prenylation and N-terminal myristoylation (only Rab5B) are indicated.
(PDF)

Figure S2 **Rooted neighbour joining phylograms of 3 major clades (A,B,C) as described in** **Figure 1** **.** Phylogenetic analysis of apicomplexan Rabs demonstrates that they belong to the major families highly conserved in other eukaryotes. Only Rab1A, Rab5B and Rab11B can be classified as alveolate or apicomplexan specific sub-class. The accession numbers can be downloaded as supporting information.
(PDF)

Figure S3 **Overview of parasite strains expressing ddFKBPmyc-tagged Rabs.** Immunofluorescence analysis and western blots of the respective Rab protein in presence (+) and absence (−) of 1 µM Shld-1. For the immunofluorescence analysis intracellular parasites expressing ddFKBPmyc-Rab1A,B,2,4,5A,5C,7,18 and Rab5B-ddFKBPHA were grown for 18 h +/− 1 µM Shld-1. The indicated Rab protein was detected by α-myc, or α-HA antibodies (green). Antibodies against the inner membrane complex (IMC) were used as control (red). Dapi was used to stain the nucleus (blue). Scale bar: 5 µm. For the western blots freshly lysed parasites treated +/− 1 µM Shld-1 for 4 hrs were used. To determine the expression of the respective Rab protein α-ddFKBP antibodies and as an internal control α-catalase antibodies were used. Asterisks (*) indicate unspecific staining.
(PDF)

Figure S4 **Localisation of Rab1A, Rab1B, Rab2, Rab4, Rab7 and Rab18.** (A–F) Intracellular parasites expressing the indicated ddFKBPmyc-Rab-construct were grown for 18 hrs in the presence of 1 µM Shld-1 prior to fixation. Co-expression of the Golgi marker GRASP-RFP, TgGalNac-YFP, the Golgi/ER marker TgERD-GFP, the Apicoplast marker FNR-RFP, or co-staining with α-proM2AP, or α-TgVP1 antibodies to label endosomal-like compartments (ELCs) were performed. The respective Rabs were detected with α-myc. Dapi is shown in blue. Scale bar: 5 µm. Co-localisations were quantified by calculating the Pearson's correlation coefficient (R). Mean values and respective standard deviation of 10–16 parasites are indicated next to the respective image.
(PDF)

Figure S5 **Localisation of Rab5A, Rab5B and Rab5C.** (A, B, D) Intracellular parasites expressing indicated ddFKBPmyc-Rab5A, 5C and Rab5B-ddFKBPHA-construct were grown for 18 hrs in presence of 1 µM Shld-1 prior to fixation. Co-expression of the Golgi marker GRASP-RFP, or co-staining with α-proM2AP, α-TgVP1or α-IMC was performed. To indicate the localisation of the respective Rab α-myc, or α-HA antibodies were used. Dapi is shown in blue. Scale bar: 5 µm. (C) Parasites co-expressing Ty-Rab5A and ddFKBPmyc-Rab5C were probed with α-Ty and α-myc antibodies. Rab5A and Rab5C show complete co-localisation. Scale bar: 5 µm. Co-localisation was quantified by calculating the Pearson's correlation coefficient (R). Mean values and respective standard deviation of 10–16 parasites are presented in a table beneath the respective image set.
(PDF)

Figure S6 **Characterisation of Rab1A.** (A) Western blot and immunofluorescence analysis of ddFKBPmyc-Rab1A(N126I) expressing parasites. For the western blot freshly lysed parasites were treated for 4 hrs +/− 1 µM Shld-1 and for the immunofluorescence analysis intracellular parasites were treated for 18 hrs +/− 1 µM Shld-1. Indicated antibodies were used. As an internal control for the western blot α-catalase antibodies were used. Dapi is shown in blue. Scale bar: 5 µm. (B) Growth analyses of the indicated parasite strains for 5 days +/− 1 µM Shld-1. The scale bar represents 1 mm. No significant growth defect was detected in parasites expressing ddFKBPmyc-Rab1A(N126I).
(PDF)

Figure S7 **Characterisation of Rab7.** (A, B) Western blot and immunofluorescence analyses of ddFKBPmyc-Rab7(G18E) and ddFKBPmyc-Rab7(N124I) expressing parasites. For the western blot freshly lysed parasites were treated for 4 h +/− 1 µM Shld-1 and for the immunofluorescence analyses intracellular parasites were treated for 18 hrs +/− 1 µM Shld-1. Indicated antibodies were used. Dapi is shown in blue. As an internal control for the western blot α-catalase antibodies were used. Scale bar: 5 µm. (C) Growth analyses of parasites expressing indicated ddFKBPRab-constructs, which were inoculated on HFF cells and cultured for 5–6 days +/− Shld-1. The scale bar represents 1 mm. (D) Immunofluorescence analysis of intracellular parasites expressing ddFKBPmyc-Rab7(G18E) and wild type parasites RH ^hxgprt−^treated for 24 hrs with 1 µM Shld-1 and probed with indicated antibodies. Dapi is shown in blue. Scale bar: 5 µm. (E) Analysis of secretory organelles (MIC3, M2AP) and ELCs (CPL, VP1) in wildtype (RH ^hxgprt−^) and ddFKBPmyc-Rab7(G18E) expressing parasites using indicated antibodies. Parasites were grown in +/− 1 µM Shld-1 for 24 hrs. Dapi is shown in blue. The scale bars represent 5 µm.
(PDF)

Figure S8 **Characterisation of Rab5B.** (A) Quantification of the localisation of rhoptry and microneme proteins in immunofluorescence analysis of parasites stably Rab5B-ddFKBPHA induced for 12, 18 and 24 hrs with 1 µM Shld-1. 300–400 PVs of three independent experiments were analysed and normalised to RH ^hxgprt−^parasites. Average (AVG) and the respective standard deviation (STD) are presented. A tendency of MIC3 secretion after 24 hrs post-induction with Shld-1 was detected, whereas M2AP, MIC2 and the rhoptry proteins ROP2-4 show no influence on the overexpression of Rab5B-ddFKBPHA. Fluorescence plus DIC images are shown (B) Western blot and immunofluorescence analysis of ddFKBPmyc-Rab5B(N152I) expressing parasites. For the western blot freshly lysed parasites were treated for 4 hrs +/− 1 µM Shld-1 and for the immunofluorescence analysis intracellular parasites were treated for 18 hrs +/− 1 µM Shld-1. Indicated antibodies were used. Dapi is shown in blue. As an internal control for the western blot α-catalase antibodies were used. Scale bar: 5 µm. (C) Growth analysis of the indicated parasite strains for 5 days in +/− 1 µM Shld-1. The scale bar represents 1 mm. (D) Immunofluorescence analysis of intracellular parasites expressing ddFKBPmyc-Rab5B(N152I) treated for 24 hrs +/− 1 µM Shld-1 and immunolabelled with the indicated antibodies. The scale bars represent 5 µm.
(PDF)

Figure S9 **Analysis of parasites overexpressing ddFKBPmyc-Rab5A and ddFKBPmyc-Rab5C.** (A) Immunofluorescence analysis of intracellular parasites expressing ddFKBPmyc-Rab5A, ddFKBPmyc-Rab5C and wild type parasites RH ^hxgprt−^treated for 24 hrs with 1 µM Shld-1 and probed with indicated antibodies (red) and Dapi (blue). For both overexpressors only MIC3, MIC8 and MIC11 are mislocalised. M2AP, MIC2 and AMA1 exhibit a normal apical localisation. (B) Replication assay of indicated parasites grown for 24 hrs in presence, or absence of 1 µM Shld-1 prior to fixation. Average number of parasites per PV was determined. (C) Egress assay of indicated parasites grown for 36 hrs +/− 1 µM Shld-1 before egress was triggered with A23187. Host cell lysis was determined 8 min after induction of egress and normalised with RH ^hxgprt−^parasites. For both overexpressors the egress is decreased. (D) Invasion assay of indicated parasites treated for 24 hrs +/− 1 µM Shld-1, scratched and inoculated on fresh HFF cells. Subsequently invasion was determined and normalised to RH ^hxgprt−^parasites. (B–D) Mean values and the respective standard deviation of three independent experiments are presented (***indicates p-value of P≤0.01, **indicates P≤0.02 and *indicates P≤0.07 in a two tailed Student's test).
(PDF)

Figure S10 **Expression of ddFKBPmyc-Rab5A(N158I) and ddFKBPmyc-Rab5C(N153I) results in a severe growth phenotype.** (A, B) Western blot and immunofluorescence analyses of parasites expressing dominant negative versions of ddFKBPmyc-Rab5A(N158I) and ddFKBPmyc-Rab5C(N153I). For the western blot freshly lysed parasites were treated for 4 hrs in presence (+), or absence (−) of 1 µM Shld-1 and for the immunofluorescence analysis intracellular parasites were treated for 18 hrs +/− 1 µM Shld-1. The corresponding Rab protein was detected by α-ddFKBP antibodies (green). As an internal control for the western blot α-catalase antibodies were used. Dapi is shown in blue. Asterisk (*) indicates an unspecific signal in the western blot. The scale bars represent 5 µm. (C) Parasites (over)-expressing indicated versions of Rab5-GTPases were inoculated on HFF cells and incubated for 5–6 days +/−1 µM Shld-1. The scale bar represents 1 mm. Overexpression of Rab5A/C and expression of dominant negative versions results in severe growth defects.
(PDF)

Figure S11 **Normal organelle formation and distribution in parasites expressing ddFKBPmyc-Rab5A(N158I).** Immunofluorescence analysis of intracellular parasites stably expressing the dominant negative ddFKBPmyc-Rab5A(N158I) and wild type parasites RH ^hxgprt−^treated for 24 hrs with 1 µM Shld-1 co-expressed with organellar markers for the apicoplast (FNR-RFP), the Golgi (GRASP-RFP), the Mitochondrion (HSP60-RFP), or co-stained with α-IMC (inner membrane complex) and α-GRA9 (dense granules) antibodies. To detect the expression of ddFKBPmyc-Rab5A(N158I) samples were additionally probed with α-myc antibodies. Dapi is stained in blue. Scale bars represent 5 µm. Expression of dominant negative ddFKBPmyc-Rab5A(N158I) shows no negative effects on all tested organelles.
(PDF)

File S1 **Overview and accession numbers of all sequences of Rab-GTPases used in the phylogenetic analysis.**
(XLS)

Table S1 **Overview of Apicomplexan Rab-GTPases.**
(PDF)

Table S2 **Summary of Rab-GTPases in** ***T.gondii***.
(PDF)

Table S3 **List of primers used in this study.**
(PDF)

Text S1 **Results and discussion of localisation analysis of Rabs.** We provide a supplementary section, discussing the results (Figure S4&5) of the localisation analysis of *T.gondii* Rab-GTPases.
(PDF)
