# Supplementary figures and images for: An Overexpression Screen of Toxoplasma gondii Rab-GTPases Reveals Distinct Transport Routes to the Micronemes
Source: PLoS Pathog. 2013 Mar 7;9(3):e1003213. doi: 10.1371/journal.ppat.1003213 (PMC3591302; doi:10.1371/journal.ppat.1003213)

Figure S1. Alignment of Rab-like proteins of *T. gondii*

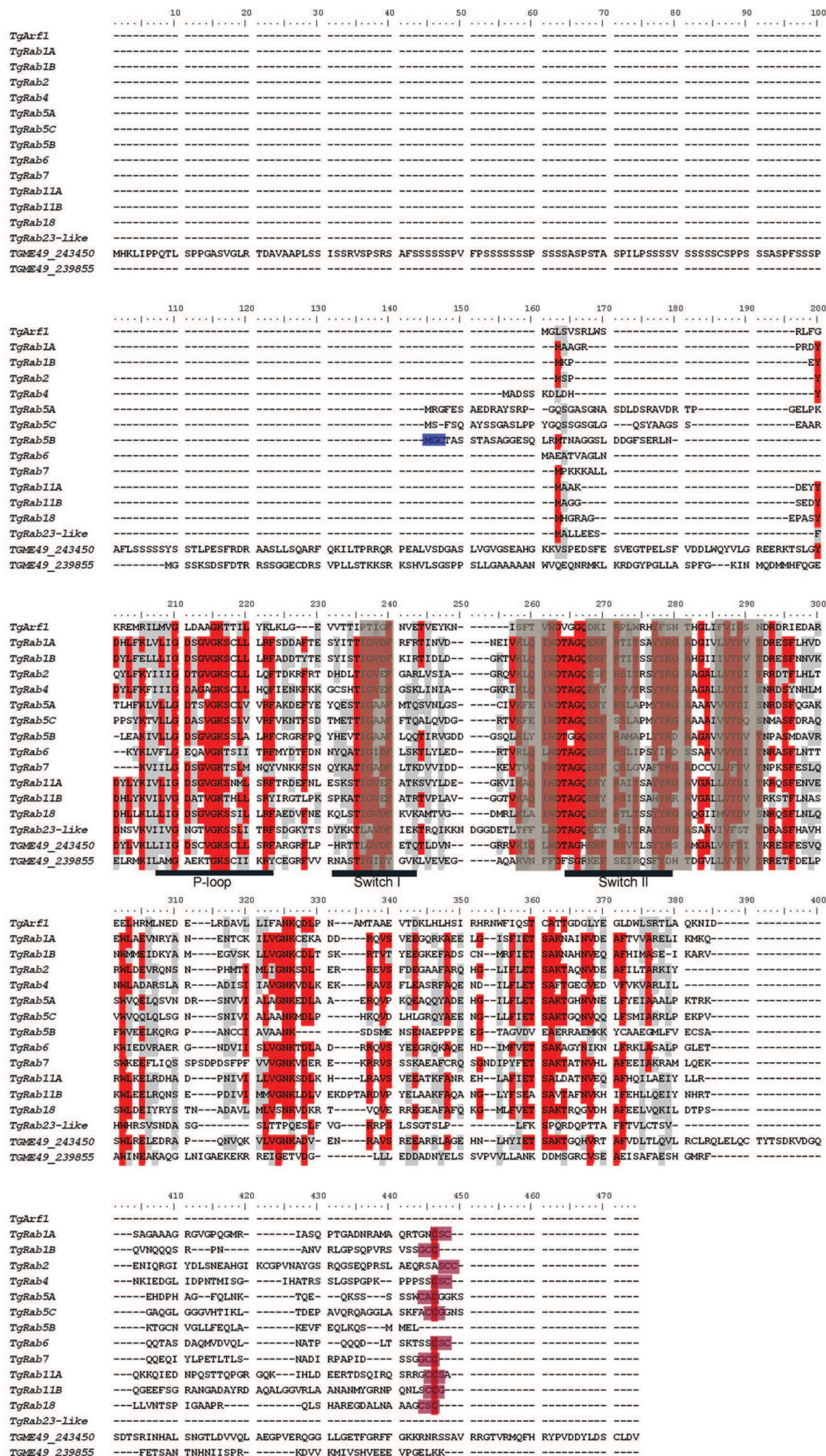

Supplement: Figure S1 — Alignment of Rab-like proteins of T. gondii . Rab consensus motifs are shaded in grey. Highly conserved regions are indicated in red (80% similarity) and grey (50% similarity). Putative motifs for C-terminal prenylation and N-terminal myristoylation (only Rab5B) are indicated. (PDF) [file ppat.1003213.s001.pdf]

**Figure S2. Rooted neighbour joining phylograms of 3 major clades (A,B,C) as described in Fig.1**

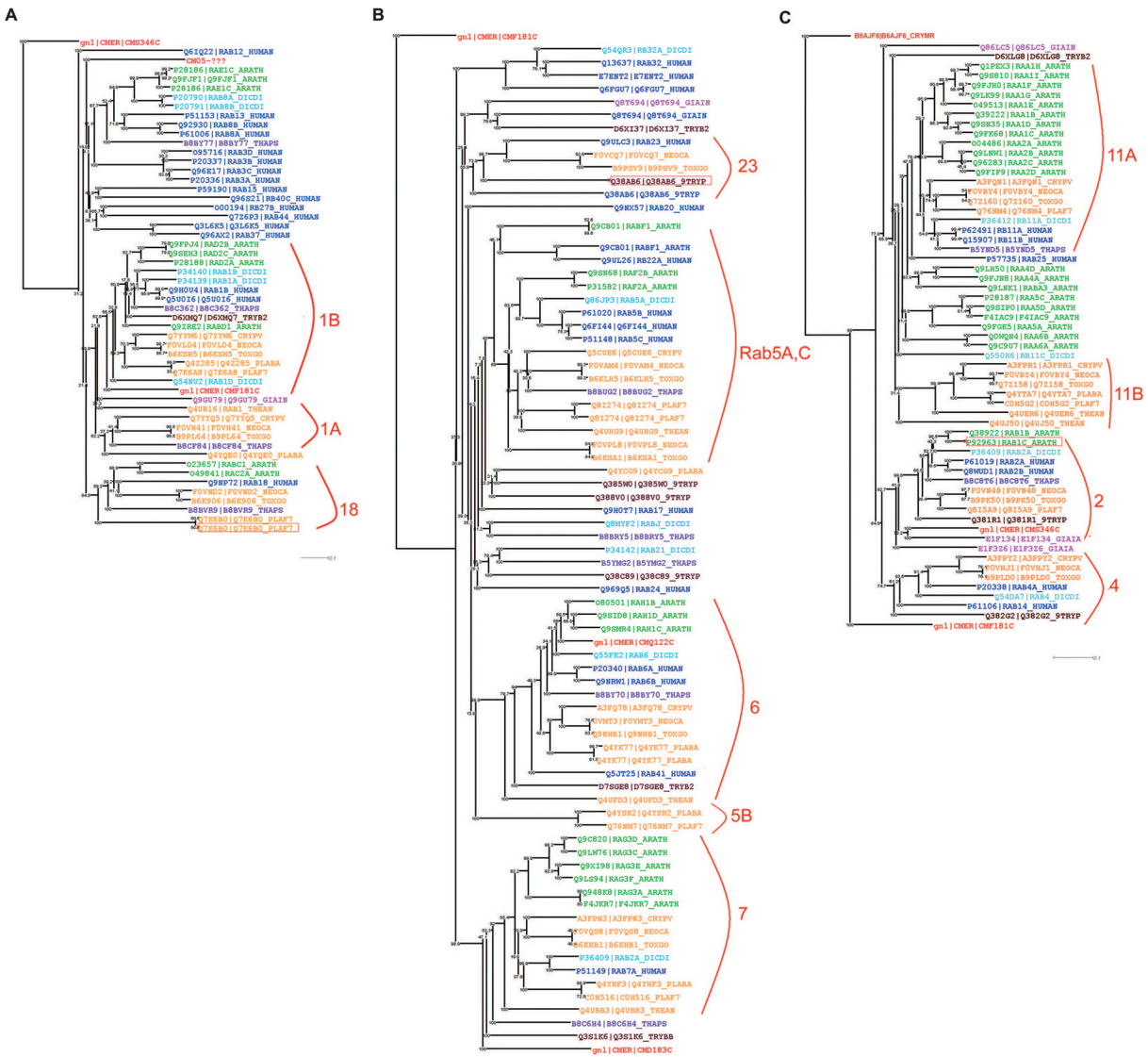

Supplement: Figure S2 — Rooted neighbour joining phylograms of 3 major clades (A,B,C) as described in Figure 1 . Phylogenetic analysis of apicomplexan Rabs demonstrates that they belong to the major families highly conserved in other eukaryotes. Only Rab1A, Rab5B and Rab11B can be classified as alveolate or apicomplexan specific sub-class. The accession numbers can be downloaded as supporting information. (PDF) [file ppat.1003213.s002.pdf]

**Figure S3. Overview of parasite strains expressing ddFKBpmyc-tagged Rab-GTPases**

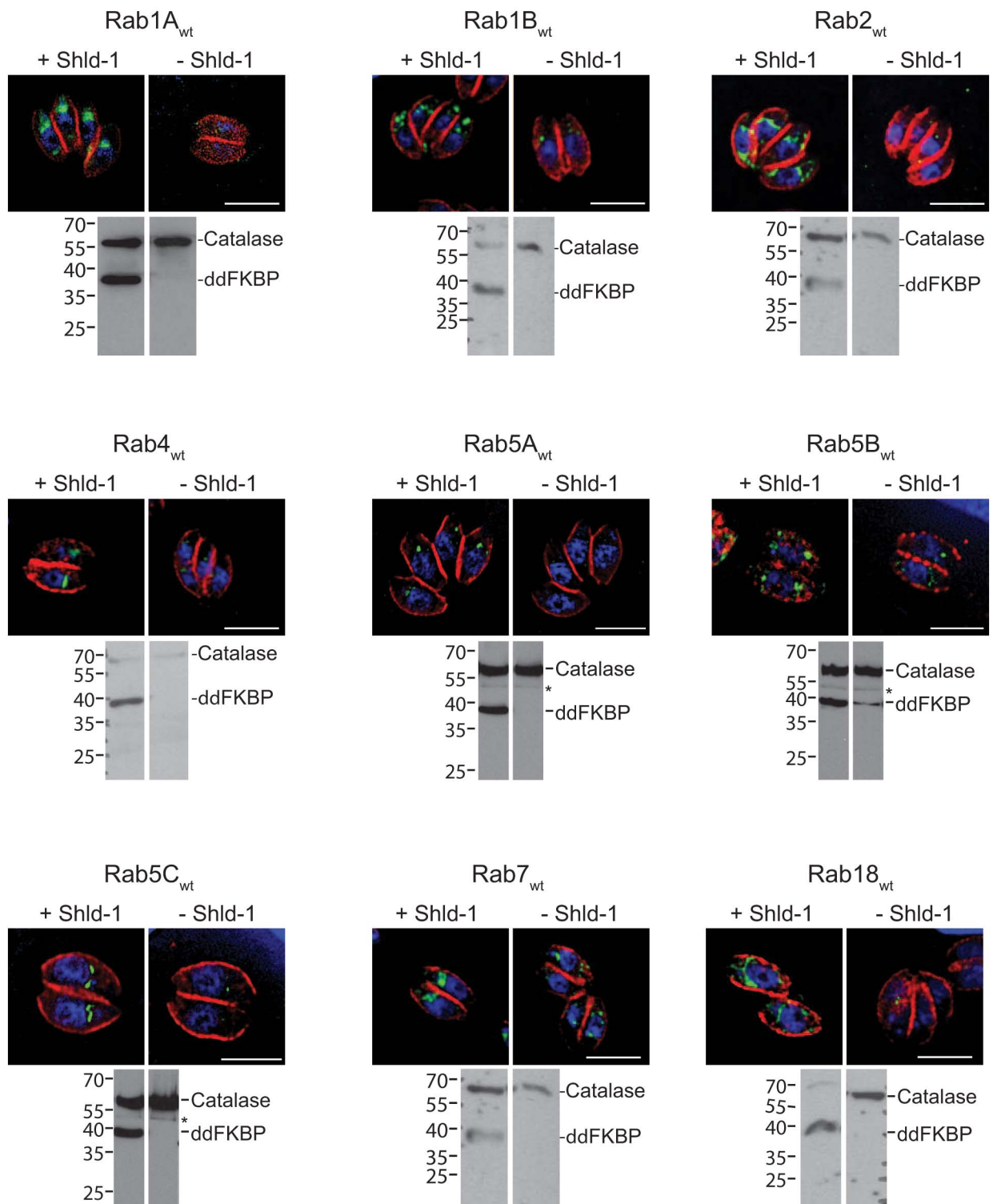

Supplement: Figure S3 — Overview of parasite strains expressing ddFKBPmyc-tagged Rabs. Immunofluorescence analysis and western blots of the respective Rab protein in presence (+) and absence (−) of 1 µM Shld-1. For the immunofluorescence analysis intracellular parasites expressing ddFKBPmyc-Rab1A,B,2,4,5A,5C,7,18 and Rab5B-ddFKBPHA were grown for 18 h +/− 1 µM Shld-1. The indicated Rab protein was detected by α-myc, or α-HA antibodies (green). Antibodies against the inner membrane complex (IMC) were used as control (red). Dapi was used to stain the nucleus (blue). Scale bar: 5 µm. For the western blots freshly lysed parasites treated +/− 1 µM Shld-1 for 4 hrs were used. To determine the expression of the respective Rab protein α-ddFKBP antibodies and as an internal control α-catalase antibodies were used. Asterisks (*) indicate unspecific staining. (PDF) [file ppat.1003213.s003.pdf]

**Figure S4. Localisation of Rab1A, Rab1B, Rab2, Rab4, Rab7 and Rab18**

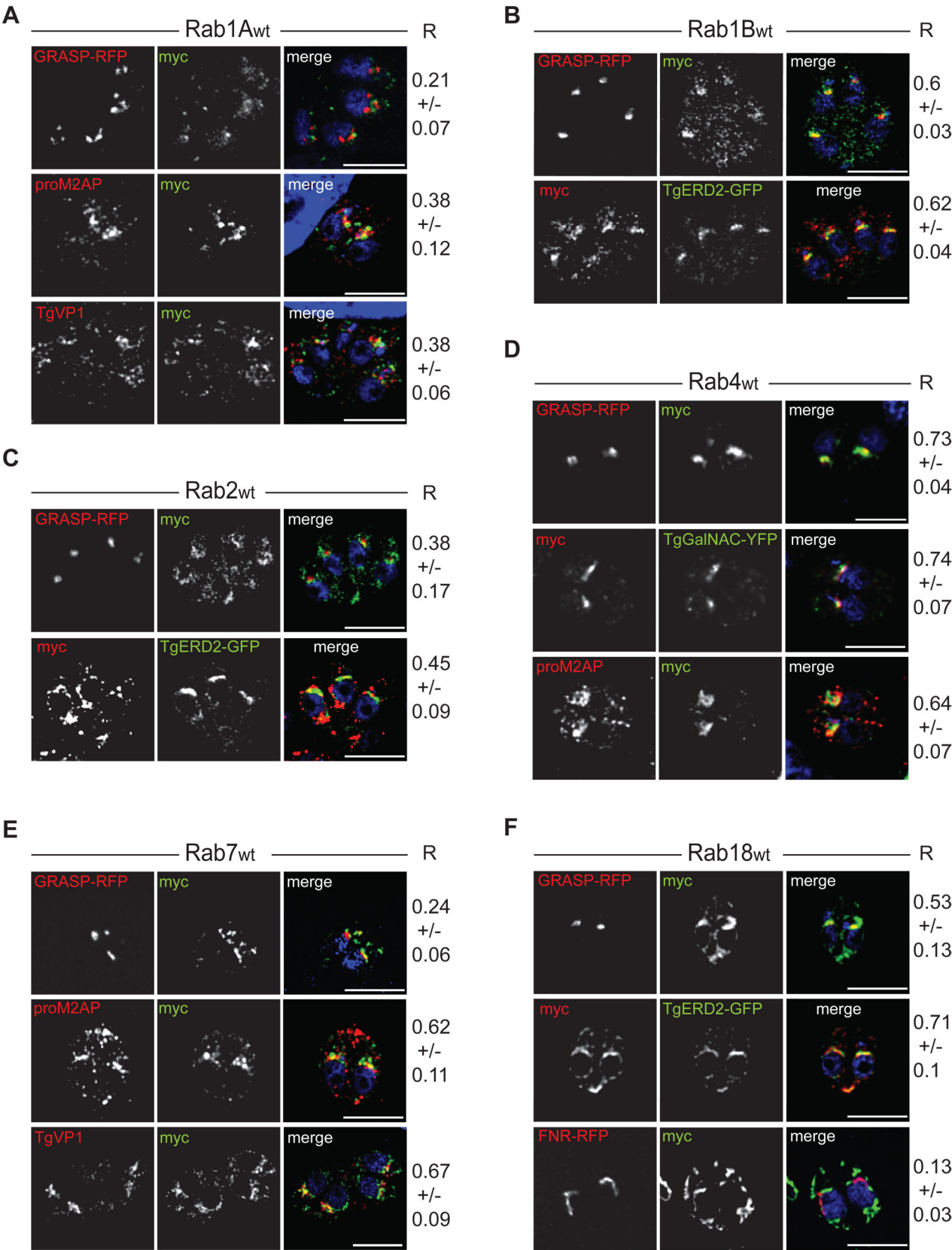

Supplement: Figure S4 — Localisation of Rab1A, Rab1B, Rab2, Rab4, Rab7 and Rab18. (A–F) Intracellular parasites expressing the indicated ddFKBPmyc-Rab-construct were grown for 18 hrs in the presence of 1 µM Shld-1 prior to fixation. Co-expression of the Golgi marker GRASP-RFP, TgGalNac-YFP, the Golgi/ER marker TgERD-GFP, the Apicoplast marker FNR-RFP, or co-staining with α-proM2AP, or α-TgVP1 antibodies to label endosomal-like compartments (ELCs) were performed. The respective Rabs were detected with α-myc. Dapi is shown in blue. Scale bar: 5 µm. Co-localisations were quantified by calculating the Pearson's correlation coefficient (R). Mean values and respective standard deviation of 10–16 parasites are indicated next to the respective image. (PDF) [file ppat.1003213.s004.pdf]

**Figure S5. Localisation of Rab5A, Rab5B and Rab5C**

**A**

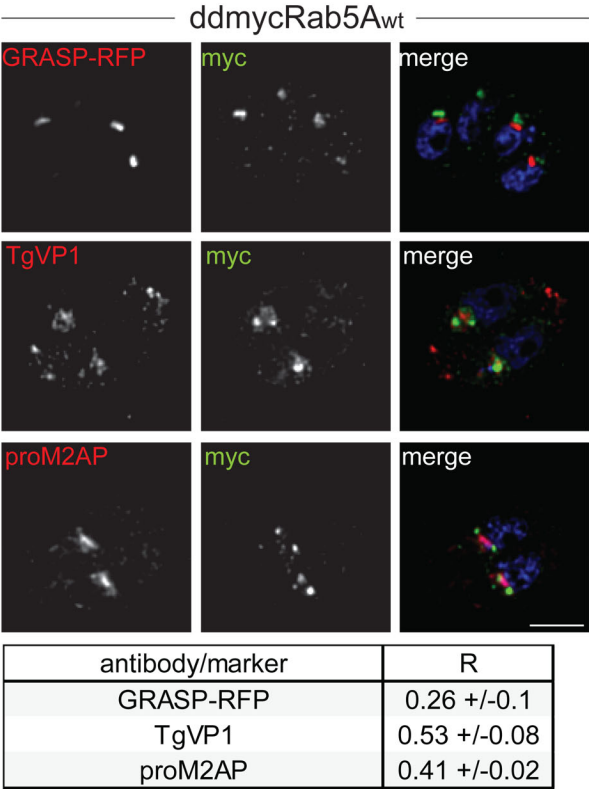

**B**

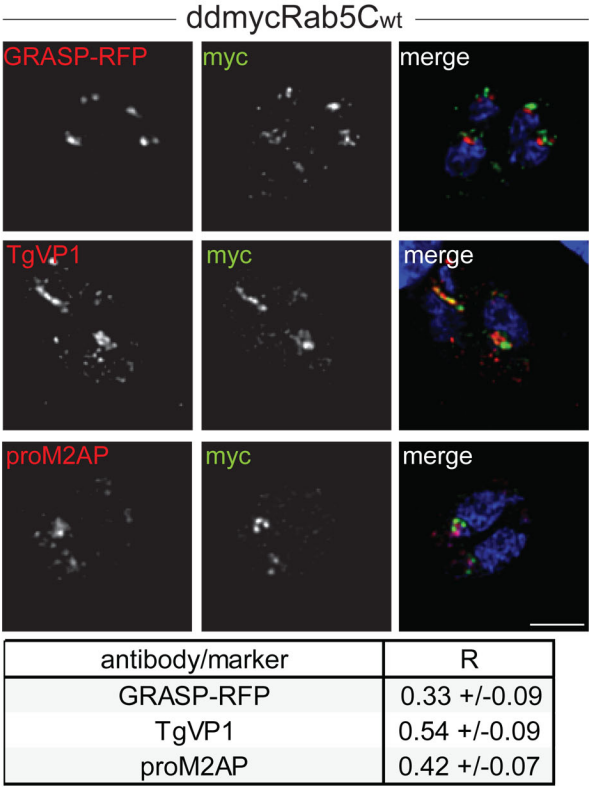

**C**

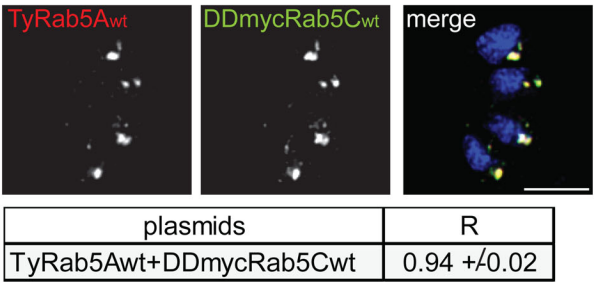

**D**

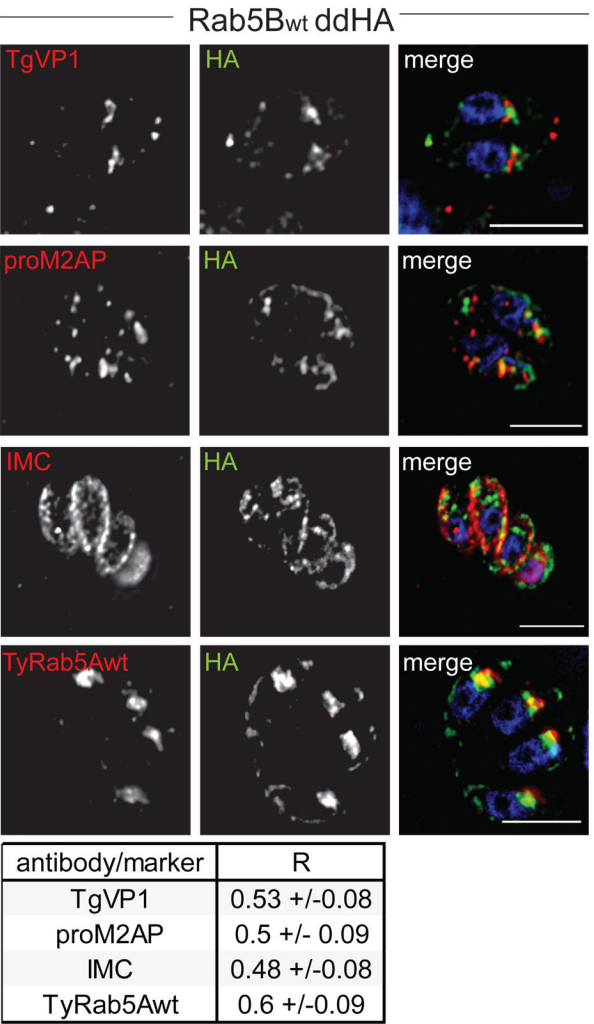

Supplement: Figure S5 — Localisation of Rab5A, Rab5B and Rab5C. (A, B, D) Intracellular parasites expressing indicated ddFKBPmyc-Rab5A, 5C and Rab5B-ddFKBPHA-construct were grown for 18 hrs in presence of 1 µM Shld-1 prior to fixation. Co-expression of the Golgi marker GRASP-RFP, or co-staining with α-proM2AP, α-TgVP1or α-IMC was performed. To indicate the localisation of the respective Rab α-myc, or α-HA antibodies were used. Dapi is shown in blue. Scale bar: 5 µm. (C) Parasites co-expressing Ty-Rab5A and ddFKBPmyc-Rab5C were probed with α-Ty and α-myc antibodies. Rab5A and Rab5C show complete co-localisation. Scale bar: 5 µm. Co-localisation was quantified by calculating the Pearson's correlation coefficient (R). Mean values and respective standard deviation of 10–16 parasites are presented in a table beneath the respective image set. (PDF) [file ppat.1003213.s005.pdf]

**Figure S6. Characterisation of Rab1A**

**A**

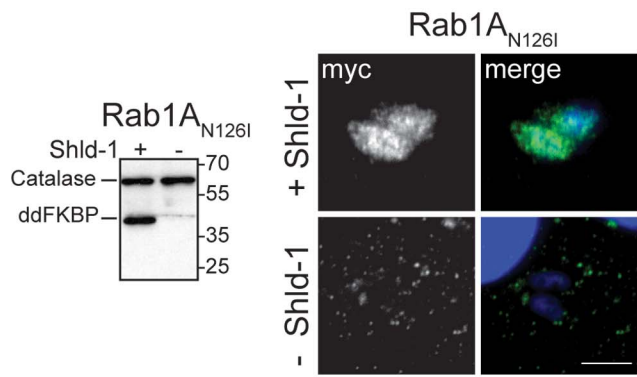

**B**

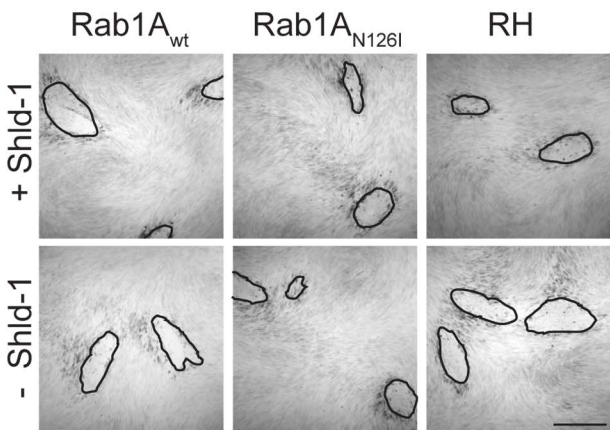

Supplement: Figure S6 — Characterisation of Rab1A. (A) Western blot and immunofluorescence analysis of ddFKBPmyc-Rab1A(N126I) expressing parasites. For the western blot freshly lysed parasites were treated for 4 hrs +/− 1 µM Shld-1 and for the immunofluorescence analysis intracellular parasites were treated for 18 hrs +/− 1 µM Shld-1. Indicated antibodies were used. As an internal control for the western blot α-catalase antibodies were used. Dapi is shown in blue. Scale bar: 5 µm. (B) Growth analyses of the indicated parasite strains for 5 days +/− 1 µM Shld-1. The scale bar represents 1 mm. No significant growth defect was detected in parasites expressing ddFKBPmyc-Rab1A(N126I). (PDF) [file ppat.1003213.s006.pdf]

Figure S7. Characterisation of Rab7

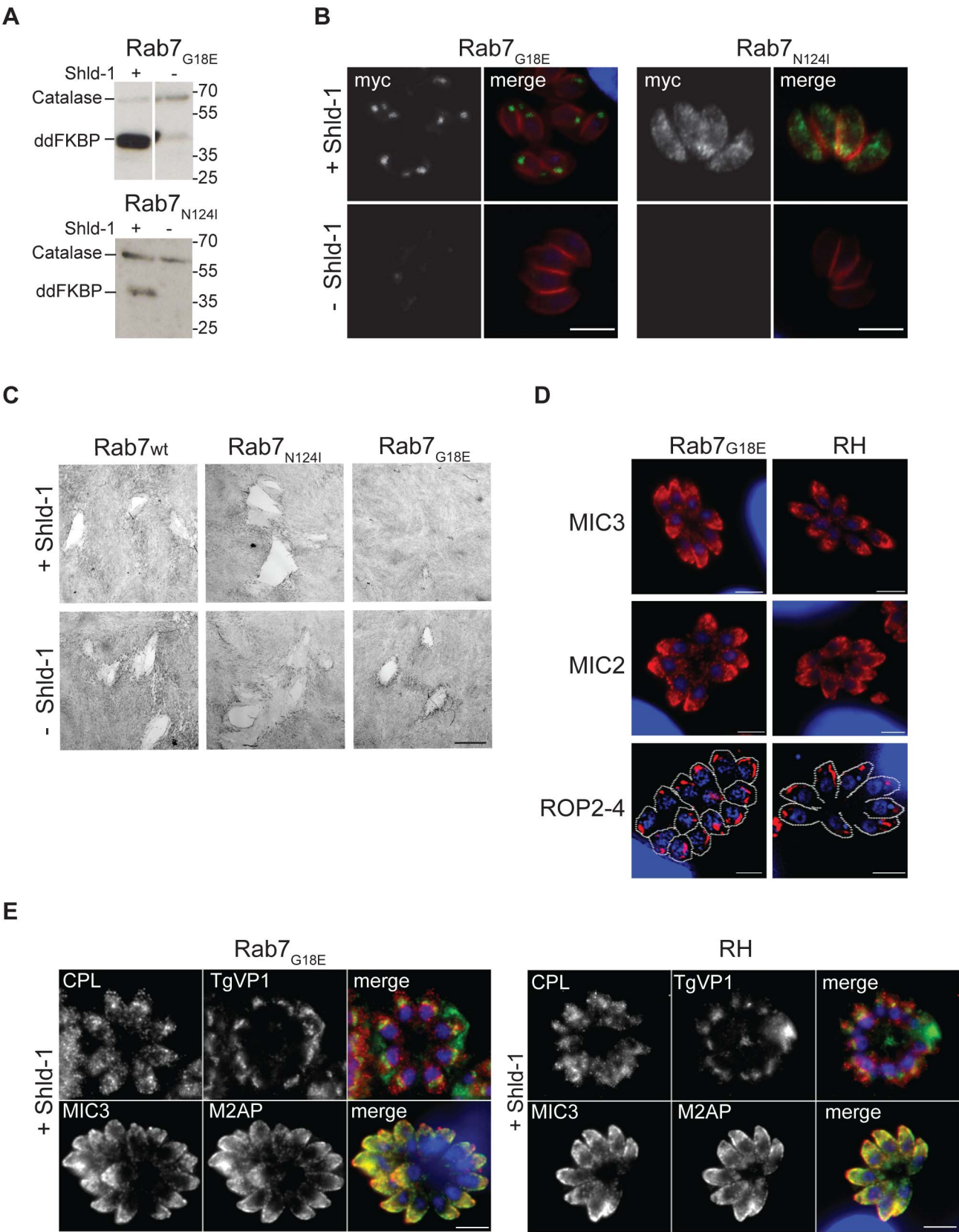

Supplement: Figure S7 — Characterisation of Rab7. (A, B) Western blot and immunofluorescence analyses of ddFKBPmyc-Rab7(G18E) and ddFKBPmyc-Rab7(N124I) expressing parasites. For the western blot freshly lysed parasites were treated for 4 h +/− 1 µM Shld-1 and for the immunofluorescence analyses intracellular parasites were treated for 18 hrs +/− 1 µM Shld-1. Indicated antibodies were used. Dapi is shown in blue. As an internal control for the western blot α-catalase antibodies were used. Scale bar: 5 µm. (C) Growth analyses of parasites expressing indicated ddFKBPRab-constructs, which were inoculated on HFF cells and cultured for 5–6 days +/− Shld-1. The scale bar represents 1 mm. (D) Immunofluorescence analysis of intracellular parasites expressing ddFKBPmyc-Rab7(G18E) and wild type parasites RH hxgprt−treated for 24 hrs with 1 µM Shld-1 and probed with indicated antibodies. Dapi is shown in blue. Scale bar: 5 µm. (E) Analysis of secretory organelles (MIC3, M2AP) and ELCs (CPL, VP1) in wildtype (RH hxgprt−) and ddFKBPmyc-Rab7(G18E) expressing parasites using indicated antibodies. Parasites were grown in +/− 1 µM Shld-1 for 24 hrs. Dapi is shown in blue. The scale bars represent 5 µm. (PDF) [file ppat.1003213.s007.pdf]

Figure S8. Characterisation of Rab5B

A

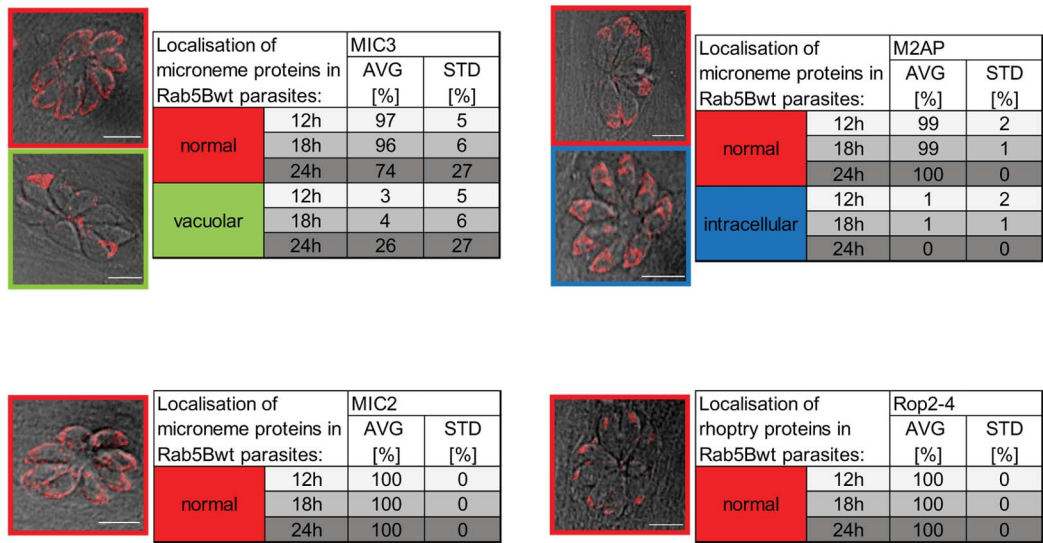

B

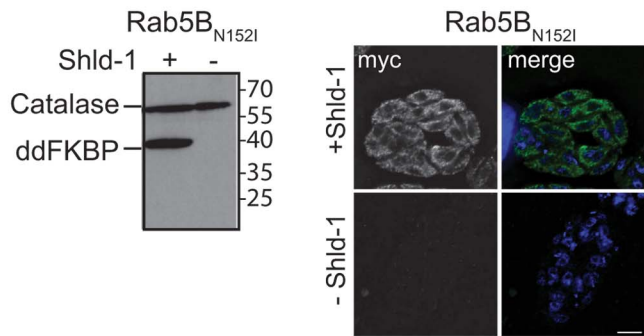

C

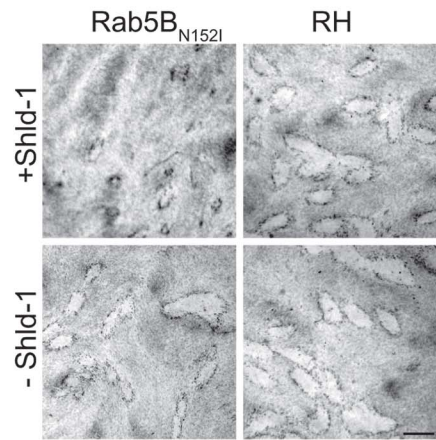

D

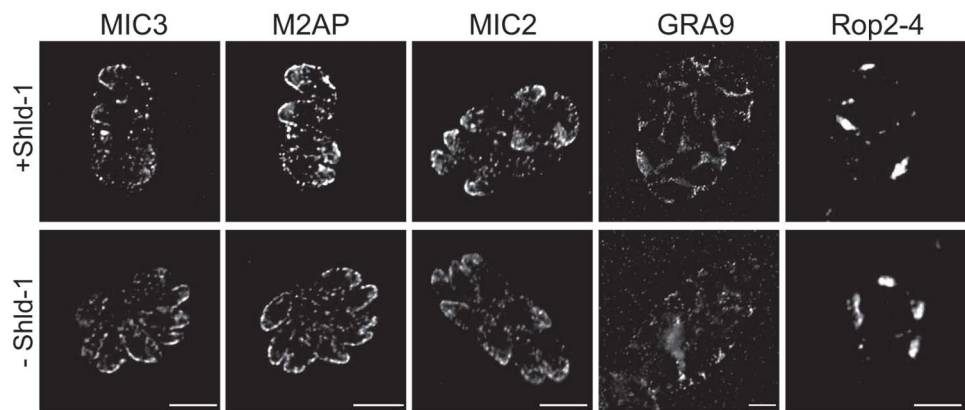

Supplement: Figure S8 — Characterisation of Rab5B. (A) Quantification of the localisation of rhoptry and microneme proteins in immunofluorescence analysis of parasites stably Rab5B-ddFKBPHA induced for 12, 18 and 24 hrs with 1 µM Shld-1. 300–400 PVs of three independent experiments were analysed and normalised to RH hxgprt−parasites. Average (AVG) and the respective standard deviation (STD) are presented. A tendency of MIC3 secretion after 24 hrs post-induction with Shld-1 was detected, whereas M2AP, MIC2 and the rhoptry proteins ROP2-4 show no influence on the overexpression of Rab5B-ddFKBPHA. Fluorescence plus DIC images are shown (B) Western blot and immunofluorescence analysis of ddFKBPmyc-Rab5B(N152I) expressing parasites. For the western blot freshly lysed parasites were treated for 4 hrs +/− 1 µM Shld-1 and for the immunofluorescence analysis intracellular parasites were treated for 18 hrs +/− 1 µM Shld-1. Indicated antibodies were used. Dapi is shown in blue. As an internal control for the western blot α-catalase antibodies were used. Scale bar: 5 µm. (C) Growth analysis of the indicated parasite strains for 5 days in +/− 1 µM Shld-1. The scale bar represents 1 mm. (D) Immunofluorescence analysis of intracellular parasites expressing ddFKBPmyc-Rab5B(N152I) treated for 24 hrs +/− 1 µM Shld-1 and immunolabelled with the indicated antibodies. The scale bars represent 5 µm. (PDF) [file ppat.1003213.s008.pdf]

**Figure S9. Analysis of parasites overexpressing ddFKBPRab5A and ddFKBPRab5C**

**A**

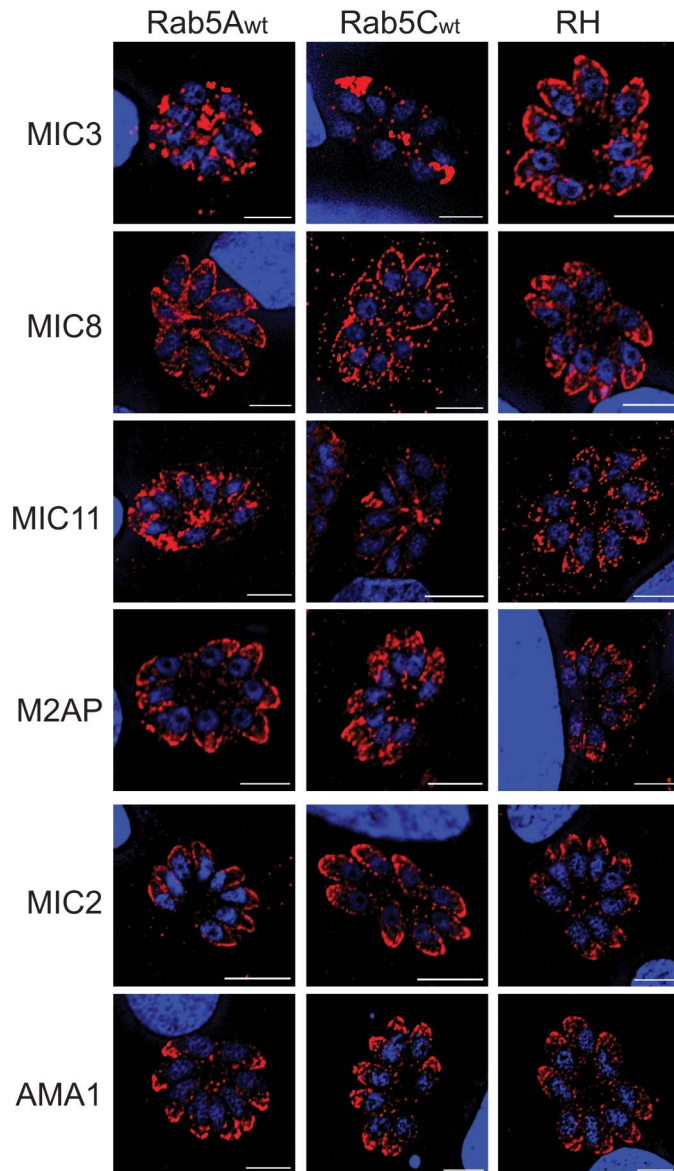

**B**

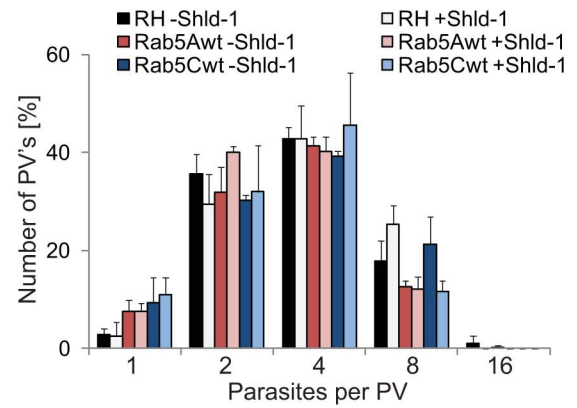

**C**

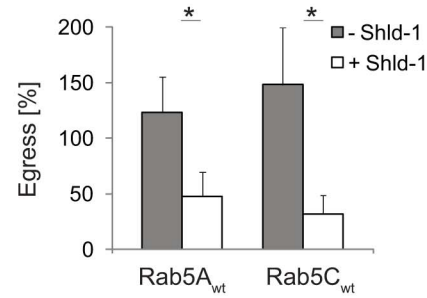

**D**

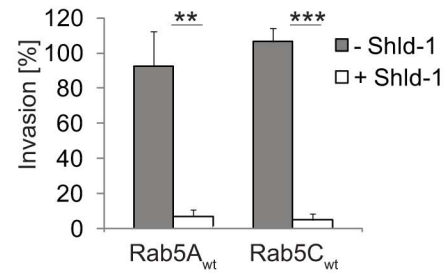

Supplement: Figure S9 — Analysis of parasites overexpressing ddFKBPmyc-Rab5A and ddFKBPmyc-Rab5C. (A) Immunofluorescence analysis of intracellular parasites expressing ddFKBPmyc-Rab5A, ddFKBPmyc-Rab5C and wild type parasites RH hxgprt−treated for 24 hrs with 1 µM Shld-1 and probed with indicated antibodies (red) and Dapi (blue). For both overexpressors only MIC3, MIC8 and MIC11 are mislocalised. M2AP, MIC2 and AMA1 exhibit a normal apical localisation. (B) Replication assay of indicated parasites grown for 24 hrs in presence, or absence of 1 µM Shld-1 prior to fixation. Average number of parasites per PV was determined. (C) Egress assay of indicated parasites grown for 36 hrs +/− 1 µM Shld-1 before egress was triggered with A23187. Host cell lysis was determined 8 min after induction of egress and normalised with RH hxgprt−parasites. For both overexpressors the egress is decreased. (D) Invasion assay of indicated parasites treated for 24 hrs +/− 1 µM Shld-1, scratched and inoculated on fresh HFF cells. Subsequently invasion was determined and normalised to RH hxgprt−parasites. (B–D) Mean values and the respective standard deviation of three independent experiments are presented (***indicates p-value of P≤0.01, **indicates P≤0.02 and *indicates P≤0.07 in a two tailed Student's test). (PDF) [file ppat.1003213.s009.pdf]

**Figure S11. Normal organelle formation and distribution in parasites expressing ddFKBPRab5A(N158I)**

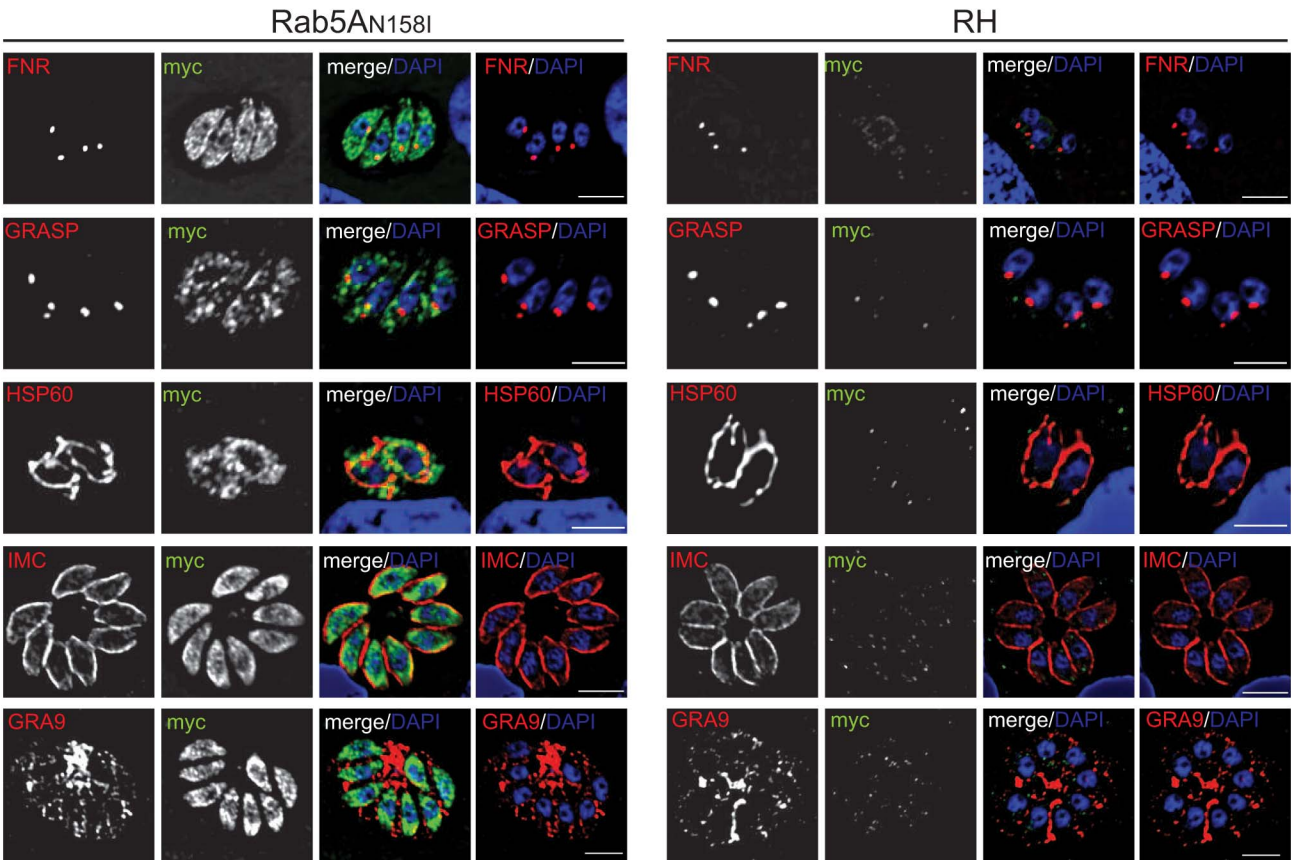

Supplement: Figure S11 — Normal organelle formation and distribution in parasites expressing ddFKBPmyc-Rab5A(N158I). Immunofluorescence analysis of intracellular parasites stably expressing the dominant negative ddFKBPmyc-Rab5A(N158I) and wild type parasites RH hxgprt−treated for 24 hrs with 1 µM Shld-1 co-expressed with organellar markers for the apicoplast (FNR-RFP), the Golgi (GRASP-RFP), the Mitochondrion (HSP60-RFP), or co-stained with α-IMC (inner membrane complex) and α-GRA9 (dense granules) antibodies. To detect the expression of ddFKBPmyc-Rab5A(N158I) samples were additionally probed with α-myc antibodies. Dapi is stained in blue. Scale bars represent 5 µm. Expression of dominant negative ddFKBPmyc-Rab5A(N158I) shows no negative effects on all tested organelles. (PDF) [file ppat.1003213.s011.pdf]
