# Supplementary material for: An Overexpression Screen of Toxoplasma gondii Rab-GTPases Reveals Distinct Transport Routes to the Micronemes
Source: PLoS Pathog. 2013 Mar 7;9(3):e1003213. doi: 10.1371/journal.ppat.1003213 (PMC3591302; doi:10.1371/journal.ppat.1003213)

Figure S10. Expression of ddFKBPmyc-Rab5A(N158I) and ddFKBPmyc-Rab5C(N153I) results in a severe growth phenotype

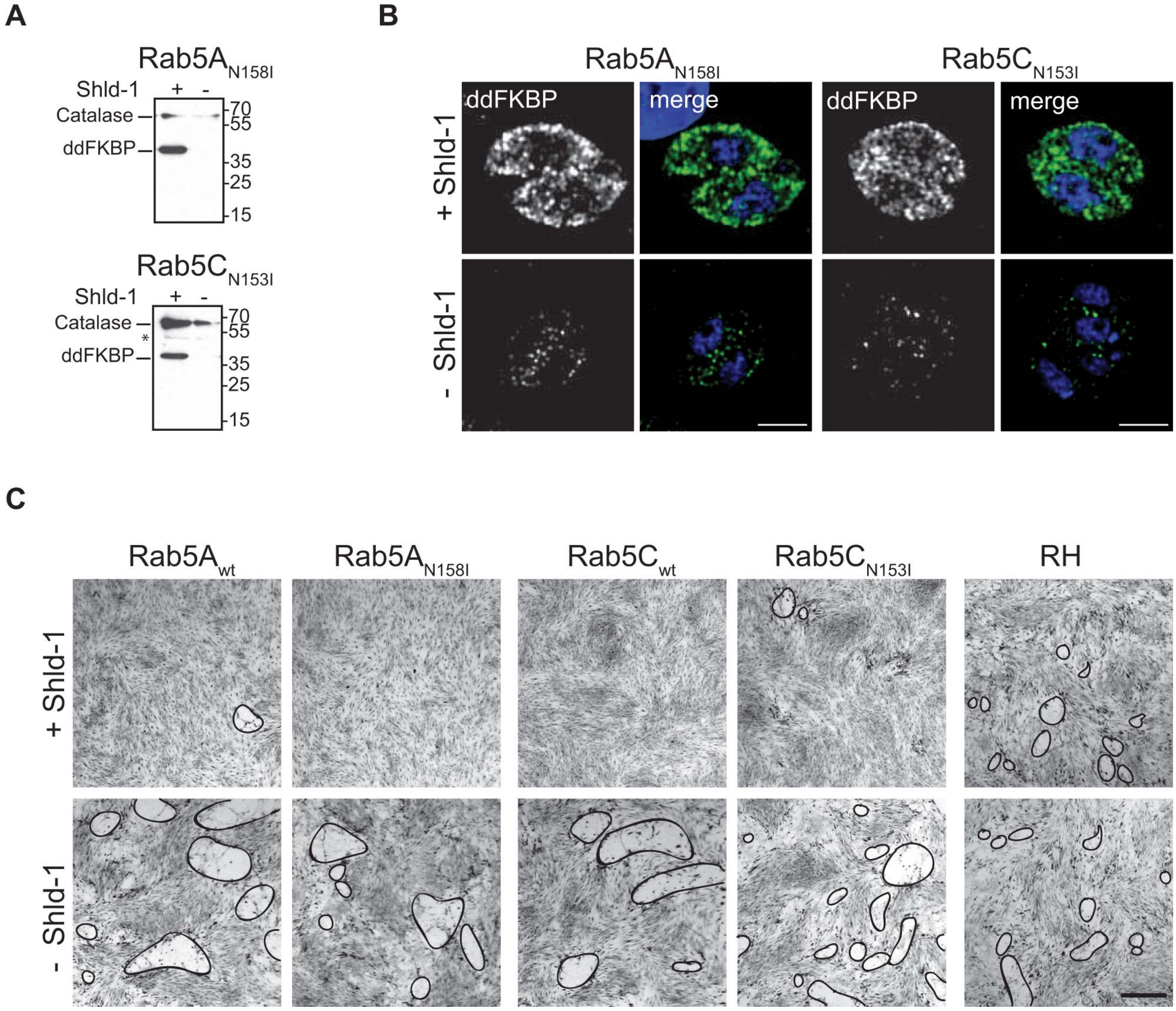

Supplement: Figure S10 — Expression of ddFKBPmyc-Rab5A(N158I) and ddFKBPmyc-Rab5C(N153I) results in a severe growth phenotype. (A, B) Western blot and immunofluorescence analyses of parasites expressing dominant negative versions of ddFKBPmyc-Rab5A(N158I) and ddFKBPmyc-Rab5C(N153I). For the western blot freshly lysed parasites were treated for 4 hrs in presence (+), or absence (−) of 1 µM Shld-1 and for the immunofluorescence analysis intracellular parasites were treated for 18 hrs +/− 1 µM Shld-1. The corresponding Rab protein was detected by α-ddFKBP antibodies (green). As an internal control for the western blot α-catalase antibodies were used. Dapi is shown in blue. Asterisk (*) indicates an unspecific signal in the western blot. The scale bars represent 5 µm. (C) Parasites (over)-expressing indicated versions of Rab5-GTPases were inoculated on HFF cells and incubated for 5–6 days +/−1 µM Shld-1. The scale bar represents 1 mm. Overexpression of Rab5A/C and expression of dominant negative versions results in severe growth defects. (PDF) [file ppat.1003213.s010.pdf]
