# Supplementary material for: An Overexpression Screen of Toxoplasma gondii Rab-GTPases Reveals Distinct Transport Routes to the Micronemes
Source: PLoS Pathog. 2013 Mar 7;9(3):e1003213. doi: 10.1371/journal.ppat.1003213 (PMC3591302; doi:10.1371/journal.ppat.1003213)
Supplement: Table S1 — Overview of Apicomplexan Rab-GTPases. (PDF) [file ppat.1003213.s013.pdf]

Table S1. Overview of Apicomplexan Rabs

Rab	T.gondii	N.caninum	C.parvum	T.parva	B.bovis	P.falciparum
**1A**	TGME49_258130	NCLIV_028240	cgd6_3830	TP04_0644	XP_001611865.1	PFE0690c
**1B**	TGME49_214770	NCLIV_051820	cgd6_3220	TP03_0775	XP_001611213.1	PFE0625w
**2**	TGME49_312050	NCLIV_055690	cgd1_2060	TP01_0877	XP_001610821.1	PFL1500w
**4**	TGME49_257340	NCLIV_029730	cgd8_1040	no	no	no
**5A**	TGME49_267810	NCLIV_038540	cgd3_3150	TP04_0575	XP_001611807.1	PFB0500c
**5B**	TGME49_207460	NCLIV_002540	no	no	no	MAL13P1.51
**5C**	TGME49_219720	NCLIV_060890	no	TP01_0639	XP_001609456.1	PFA0335w
**6**	TGME49_310460	NCLIV_054540	cgd2_1940	TP02_0799	XP_001611576.1	PF11_0461
**7**	TGME49_248880	NCLIV_065010	cgd7_1680	TP03_0666	XP_001611307.1	PFI0155c
**11A**	TGME49_289680	NCLIV_041930	cgd4_320	TP01_1204	XP_001610582.1	PF13_0119
**11B**	TGME49_320480	NCLIV_009790	cgd7_4380	TP02_0559	XP_001610116.1	MAL13P1.205
**18**	TGME49_313190	NCLIV_056520	no	no	no	PF08_0110
**23**	TGME49_283530	NCLIV_015390	no	no	no	no
**8/10 like**	TGME49_243450	NCLIV_018180	no	no	no	no
**Rab-like**	TGME49_239855	no	no	no	no	no
