# Supplementary material for: An Overexpression Screen of Toxoplasma gondii Rab-GTPases Reveals Distinct Transport Routes to the Micronemes
Source: PLoS Pathog. 2013 Mar 7;9(3):e1003213. doi: 10.1371/journal.ppat.1003213 (PMC3591302; doi:10.1371/journal.ppat.1003213)
Supplement: Table S2 — Summary of Rab-GTPases in T.gondii. (PDF) [file ppat.1003213.s014.pdf]

Table S2. Summary of Rabs in *T.gondii*

Rab	*T.gondii*	*C-terminal targetting*	*N-terminal Myristoylation*	*Expression evidence*	*Localisation*	*Reference*
**1A**	TGME49_258130	Yes	No	Yes	Golgi, Endosomal	this study
**1B**	TGME49_214770	Yes	No	Yes	ER/Golgi	this study
**2**	TGME49_312050	Yes	No	yes	ER/Golgi	this study
**4**	TGME49_257340	Yes	No	yes	Golgi	this study
**5A**	TGME49_267810	Yes	No	yes	Endosomal	Robibaro et al., 2002
**5B**	TGME49_207460	no	Yes	yes	Endosomal	this study
**5C**	TGME49_219720	Yes	No	yes	Endosomal	This study
**6**	TGME49_310460	Yes	No	yes	Golgi	Stedman et al., 2003
**7**	TGME49_248880	Yes	No	Yes	Endosomal	Parussini et al., 2010 Miranda et al., 2010
**11A**	TGME49_289680	Yes	No	Yes	IMC, endosomal	Agop-Nersesian et al., 2009
**11B**	TGME49_320480	Yes	No	Yes	Golgi, IMC	Agop-Nersesian et al., 2010
**18**	TGME49_313190	Yes	No	Yes	ER/Golgi	This study
**23**	TGME49_283530	no	No	No	n.d.	
**8/10 like**	TGME49_243450	no	No	yes	n.d.	
**Rab-like**	TGME49_239855	No	No	No	n.d.	
