# Supplementary material for: An Overexpression Screen of Toxoplasma gondii Rab-GTPases Reveals Distinct Transport Routes to the Micronemes
Source: PLoS Pathog. 2013 Mar 7;9(3):e1003213. doi: 10.1371/journal.ppat.1003213 (PMC3591302; doi:10.1371/journal.ppat.1003213)
Supplement: Table S3 — List of primers used in this study. (PDF) [file ppat.1003213.s015.pdf]

Table S3. List of Primers used in this study. Light grey backgrounds indicate forward primer and white backgrounds indicate reverse primer.

construct	Primer	RS
ddmycRab1A$_{wt}$-HXGPRT	CCATGCATGCGGCAGGCAGACCACGG	NsiI/PacI
	GCGTTAATTAACATGAACAGTTGCCAGTCCGCTGTGCCATAGCCCGGTTGTCAGCAC	
ddmycRab1A$_{N126I}$-HXGPRT	TCGTCTGCCTTCTCGCATTTGATTCCTACGAGGATCTTGC	
ddmycRab1B$_{wt}$-HXGPRT	GCGATGCATAAGCCTGAATACGACTATCTTTTCAAGCTGCTTCTCATTGGCGACTC	NsiI/PacI
	GCGTTAATTAACAACAACCCGAAGAGACGCTGCGAACCGGCTGG	
ddmycRab2$_{wt}$-HXGPRT	GCGATGCATATGCCGTACCAGTATCTCTTCAAGTATATCATCA	NsiI/PacI
	GCGTTAATTAACAGCAACTTGCAGACCGCTG	
ddmycRab4$_{wt}$-HXGPRT	GCGATGCATGACTCCAGCAAGGACCTG	NsiI/PacI
	GCGTTAATTAACACGAGCAACTCGATGGCGG	
ddmycRab5A$_{wt}$-HXGPRT	GCGATGCATGGTTTCGAATCTGCTGAGG	NsiI/PacI
	GCGTTAATTAACTTTTGCCTCCACATGCACACC	
TyRab5A$_{wt}$	GCGATGCATGGTTTCGAATCTGCTGAGG	EcoRI/NsiI
	GCGTTAATTAACTTTTGCCTCCACATGCACACC	
ddmycRab5A$_{N158I}$-HXGPRT	CGCGGGGATCAAAGAGG	
ddmycRab5B$_{wt}$-HXGPRT	GCGATGCATGGATGCACCGCGAGCTCCAC	NsiI/PacI
	GCGTTAATTAATCACAACTCCATCATGCTCTGCTTCAGC	
ddmycRab5B$_{N152I}$-HXGPRT	GCTGCGATCAAGAGCG	
ddmycRab5C$_{wt}$-HXGPRT	GCGATGCATTCTTTCTCGCAAGCTTACAGTTC	NsiI/PacI
	GCGTTAATTAATCAACTGTTTCCGCCGCAAC	
ddmycRab5C$_{N153I}$-HXGPRT	GCAGCCATCAAGATGG	
ddmycRab7$_{wt}$-HXGPRT	GCGATGCATCCCAAGAAGAAGGCTCTCTTGAAAG	NsiI/PacI
	GCGATGCATCCCAAGAAGAAGGCTCTCTTG	
ddmycRab7$_{N125I}$-HXGPRT	CGTTGGCATCAAAGTCG	
ddmycRab7$_{G18E}$-HXGPRT	GCGATGCATCCCAAGAAGAAGGCTCTCTTGAAAGTCATCATCCTCGGGGACAGCGAGG	NsiI/PacI
	TAGGCAAGACCTCGCTGATGAACCAGT	
ddmycRab18$_{wt}$-HXGPRT	GCGATGCATGGTCGCGCAGGA	NsiI/PacI
	GCGTTAATTAACAGGAACACCCGGCGG	
