# Supplementary material for: An Overexpression Screen of Toxoplasma gondii Rab-GTPases Reveals Distinct Transport Routes to the Micronemes
Source: PLoS Pathog. 2013 Mar 7;9(3):e1003213. doi: 10.1371/journal.ppat.1003213 (PMC3591302; doi:10.1371/journal.ppat.1003213)
Supplement: Text S1 — Results and discussion of localisation analysis of Rabs. We provide a supplementary section, discussing the results (Figure S4&5) of the localisation analysis of T.gondii Rab-GTPases. (PDF) [file ppat.1003213.s016.pdf]

List of supplementary information:

- **Result and Discussion of localisation analysis of Rabs**
- **FigureS1. Alignment of Rab-like proteins of *T. gondii.***
- **FigureS2. Rooted neighbour joining phylograms of 3 major clades (A,B,C) as described in Figure 1**
- **FigureS3. Overview of parasite strains expressing ddFKBPmyc-tagged Rabs**
- **FigureS4: Localisation of Rab1A, Rab1B, Rab2, Rab4, Rab7 and Rab18**
- **FigureS5: Localisation of Rab5A, Rab5B and Rab5C**
- **FigureS6. Characterisation of Rab1A**
- **FigureS7. Characterisation of Rab7**
- **FigureS8. Characterisation of Rab5B**
- **FigureS9. Analysis of parasites overexpressing ddFKBPmyc-Rab5A and ddFKBPmyc-Rab5C**
- **FigureS10. Expression of ddFKBPmyc-Rab5A(N158I) and ddFKBPmyc-Rab5C(N153I) results in a severe growth phenotype**
- **Figure S11. Normal organelle formation and distribution in parasites expressing ddFKBPmyc-Rab5A(N158I)**
- **Table S1. Overview of Apicomplexan Rabs**
- **Table S2. Summary of Rabs in *T.gondii***
- **Table S3. List of Primers used in this study**
- **File S1.xls Overview and accession numbers of all sequences of Rabs used in the phylogenetic analysis**

Result and Discussion of localisation analysis of Rabs

In order to identify Rabs that are involved in vesicular transport to the unique secretory organelles of *Toxoplasma gondii* we performed an overexpression screen, where each Rab has been fused to ddFKBP-myc [1], that allows rapid and tuneable regulation of protein levels.

While overexpression phenotypes can be expected at high concentrations of the inducer Shield-1, localisation analysis of the protein of interest can be performed at low concentrations and in some cases even in absence of inducer. Using this approach we found that all analysed Rabs are localised to the early-late secretory system of the parasite, but not to the apical secretory organelles.

We found that Rab1B and 18 localise predominantly to organelles of the early secretory pathway, the ER and the Golgi (Figure 1B,Figure S4B,F) as demonstrated by co-localisation with the marker proteins TgERD2 and GRASP [2]. However Rab 2 shows a tendency of accumulation within the ER/Golgi region (Figure 1B, Figure S4C), but the colocalisation is not as clear as it is for Rab1B and 18. This could suggest that Rab1B and Rab2 have a similar role in the transport of vesicles between the ER and the Golgi, as observed in other eukaryotes [3,4], but a different function within apicomplexans for Rab2 cannot be ruled out. Rab18 has been identified in several eukaryotic lineages, indicating that this Rab was present in the LCEA [5]. However, in contrast to Rab1 and 2 it has been lost in several species, including some apicomplexan parasites, where it is missing in case of *Cryptosporidium, Theileria* and *Babesia* [6]. Rab18 has been implicated in diverse roles, including ER-Golgi-traffic [7,8], formation of lipid droplets [9,10] or regulation of secretion in neuroendocrine cells [11], indicating that this protein does not show a strict functional conservation.

Rab4 can be identified in diverse eukaryotic lineages, but have been lost on several occasions [12]. Similarly, in apicomplexans Rab4 is present in *Toxoplasma* and *Cryptosporidium* but absent in *Plasmodium* and *Theileria* [6]. Rab4 had been first characterised in human cells and shown to be essential for endocytosis and the formation of early endosomes [13]. A similar role for Rab4 has been demonstrated in diverse eukaryotes, including protozoan parasites such as *Trypanosoma brucei*, where it plays an important role in endocytic recycling [14]. In contrast we found that *T.gondii* Rab4 is almost exclusively localised to the Golgi (Figure 1B, S4D), as indicated by co-localisation with GRASP-RFP and UDP-*N*-acetyl-D-galactosamine:polypeptide *N*-acetylgalactosaminyltransferase T1 (TgGalNac) fused to yellow fluorescent protein (YFP, Nishi and Roos, unpublished) (Figure 1B,Figure S4D). On some occasions we also found a partial co-localisation of Rab4 with proM2AP [15], a marker for what we call, endosomal- like compartments (Figure S4D). Post-Golgi organelles, where

TgVP1, proM2AP and TgCPL are localised are often assumed to be early or late endosomes, lysosome or other organelles like VAC or plant-like vacuole. Since no evidence exist to prove this, we prefere to call these compartments endosomal-like compartments (ELCs).

A recent phylogenetic analysis suggests that apicomplexan Rab1A defines a unique paralog shared by alveolates [16] and our phylogenetic analysis supports this view (Figure 1A,Figure S2). When we analysed parasites expressing a ddFKBPmyc-tagged version of Rab1A, we found this protein concentrated within the post-Golgi region as indicated by co-localisation with the Golgi marker GRASP-RFP and ELC marker proM2AP and TgVP1. Due to this highly dynamic location of Rab1A we cannot define a compartment, where exactly this GTPase is localised. (Figure S4A).

Rab7 has been previously localised to the ELCs in *T. gondii* [17,18]. In our study we confirmed partial co-localisation of Rab7 with ELCs (proM2AP and TgVP1) (Figure 1B,Figure S4E) , consistent with a conserved role of Rab7 in trafficking to endosomes [19]

Three Rab5-GTPases can be identified in the genome of apicomplexan parasites (Figure 1A). While Rab5A and Rab5C appear to be derived from a lineage specific gene duplication event (Figure 1A,Figure S2), Rab5B belongs to a unique class that is only conserved in apicomplexan parasites. Interestingly this protein lacks the typical prenylation motif at the C-terminus. Instead a potential myristoylation motif at the N-terminus can be identified (Figure S1). Therefore we tagged Rab5B C-terminally with ddFKBPHA for localisation studies and found that this protein showed a concentration at ELCs (Figure 1B, Figure S5D) and to a lesser extent at the surface of the parasite, possibly the inner membrane complex (IMC) (Figure S5D). Consistent with earlier studies [20], we identified Rab5A at ELCs and found an identical location for Rab5C (Figure 1B,Figure S5A,B,C), indicating a role of the two Rabs in the organisation and function of the ELCs.

Although the analysis of the localisation of Rabs is a first step to obtain information about their individual function, we would like to mention that without a more detailed analysis (as presented for Rab5A and Rab5C), we cannot draw any firm conclusions about their role. Although we found that overexpression of Rab2, Rab4 and Rab5B is not tolerated by the parasite, we were unable to identify a clear phenotypic consequence (apart from parasite death). Similarly we were unable to pinpoint the consequence of dominant active expression of Rab7(G18E) (see Figure S7).

Furthermore, we do not suggest that a particular Rab is not essential in case no overexpression phenotype can be observed (as in case of Rab18). Future studies using conditional knockout technologies should be employed to further characterise this important protein family.

References

1. Herm-Gotz A, Agop-Nersesian C, Munter S, Grimley JS, Wandless TJ, et al. (2007) Rapid control of protein level in the apicomplexan Toxoplasma gondii. Nat Methods 4: 1003-1005.
2. Pfluger SL, Goodson HV, Moran JM, Ruggiero CJ, Ye X, et al. (2005) Receptor for retrograde transport in the apicomplexan parasite Toxoplasma gondii. Eukaryot Cell 4: 432-442.
3. Dhir V, Goulding D, Field MC (2004) TbRAB1 and TbRAB2 mediate trafficking through the early secretory pathway of Trypanosoma brucei. Mol Biochem Parasitol 137: 253-265.
4. Tisdale EJ, Bourne JR, Khosravi-Far R, Der CJ, Balch WE (1992) GTP-binding mutants of rab1 and rab2 are potent inhibitors of vesicular transport from the endoplasmic reticulum to the Golgi complex. J Cell Biol 119: 749-761.
5. Elias M, Brighouse A, Gabernet-Castello C, Field MC, Dacks JB (2012) Sculpting the endomembrane system in deep time: high resolution phylogenetics of Rab GTPases. J Cell Sci 125: 2500-2508.
6. Langsley G, van Noort V, Carret C, Meissner M, de Villiers EP, et al. (2008) Comparative genomics of the Rab protein family in Apicomplexan parasites. Microbes Infect 10: 462-470.
7. Dejgaard SY, Murshid A, Erman A, Kizilay O, Verbich D, et al. (2008) Rab18 and Rab43 have key roles in ER-Golgi trafficking. J Cell Sci 121: 2768-2781.
8. Jeffries TR, Morgan GW, Field MC (2002) TbRAB18, a developmentally regulated Golgi GTPase from Trypanosoma brucei. Mol Biochem Parasitol 121: 63-74.
9. Martin S, Driessen K, Nixon SJ, Zerial M, Parton RG (2005) Regulated localization of Rab18 to lipid droplets: effects of lipolytic stimulation and inhibition of lipid droplet catabolism. J Biol Chem 280: 42325-42335.
10. Ozeki S, Cheng J, Tauchi-Sato K, Hatano N, Taniguchi H, et al. (2005) Rab18 localizes to lipid droplets and induces their close apposition to the endoplasmic reticulum-derived membrane. J Cell Sci 118: 2601-2611.
11. Vazquez-Martinez R, Cruz-Garcia D, Duran-Prado M, Peinado JR, Castano JP, et al. (2007) Rab18 inhibits secretory activity in neuroendocrine cells by interacting with secretory granules. Traffic 8: 867-882.
12. Brighouse A, Dacks JB, Field MC (2010) Rab protein evolution and the history of the eukaryotic endomembrane system. Cell Mol Life Sci 67: 3449-3465.
13. van der Sluijs P, Hull M, Webster P, Male P, Goud B, et al. (1992) The small GTP-binding protein rab4 controls an early sorting event on the endocytic pathway. Cell 70: 729-740.
14. Hall BS, Pal A, Goulding D, Field MC (2004) Rab4 is an essential regulator of lysosomal trafficking in trypanosomes. J Biol Chem 279: 45047-45056.
15. Harper JM, Huynh MH, Coppens I, Parussini F, Moreno S, et al. (2006) A cleavable propeptide influences Toxoplasma infection by facilitating the trafficking and secretion of the TgMIC2-M2AP invasion complex. Mol Biol Cell 17: 4551-4563.
16. Elias M, Patron NJ, Keeling PJ (2009) The RAB family GTPase Rab1A from Plasmodium falciparum defines a unique paralog shared by chromalveolates and rhizaria. J Eukaryot Microbiol 56: 348-356.
17. Miranda K, Pace DA, Cintron R, Rodrigues JC, Fang J, et al. (2010) Characterization of a novel organelle in Toxoplasma gondii with similar composition and function to the plant vacuole. Mol Microbiol 76: 1358-1375.
18. Parussini F, Coppens I, Shah PP, Diamond SL, Carruthers VB (2010) Cathepsin L occupies a vacuolar compartment and is a protein maturase within the endo/exocytic system of Toxoplasma gondii. Mol Microbiol 76: 1340-1357.
19. Vanlandingham PA, Ceresa BP (2009) Rab7 regulates late endocytic trafficking downstream of multivesicular body biogenesis and cargo sequestration. J Biol Chem 284: 12110-12124.
20. Robibaro B, Stedman TT, Coppens I, Ngo HM, Pypaert M, et al. (2002) Toxoplasma gondii Rab5 enhances cholesterol acquisition from host cells. Cell Microbiol 4: 139-152.

Supplementary Figure Legends.

Figure S1: Alignment of Rab-like proteins of *T. gondii.* Rab consensus motifs are shaded in grey. Highly conserved regions are indicated in red (80% similarity) and grey (50% similarity). Putative motifs for C-terminal prenylation and N-terminal myristoylation (only Rab5B) are indicated.

Figure S2: Rooted neighbour joining phylograms of 3 major clades (A,B,C) as described in Figure 1 Phylogenetic analysis of apicomplexan Rabs demonstrates that they belong to the major families highly conserved in other eukaryotes. Only Rab1A, Rab5B and Rab11B can be classified as alveolate or apicomplexan specific sub-class. The accession numbers can be downloaded as supporting information.

Figure S3: Overview of parasite strains expressing ddFKBPmyc-tagged Rabs. Immunofluorescence analysis and western blots of the respective Rab protein in presence (+) and absence (-) of 1 µM Shld-1. For the immunofluorescence analysis intracellular parasites expressing ddFKBPmyc-Rab1A,B,2,4,5A,5C,7,18 and Rab5B-ddFKBPHA were grown for 18 h +/- 1 µM Shld-1. The indicated Rab protein was detected by α-myc, or α-HA antibodies (green). Antibodies against the inner membrane complex (IMC) were used as control (red). Dapi was used to stain the nucleus (blue). Scale bar: 5 µm. For the western blots freshly lysed parasites treated +/- 1 µM Shld-1 for 4hrs were used. To determine the expression of the respective Rab protein α-ddFKBP antibodies and as an internal control α-catalase antibodies were used. Asterisks (*) indicate unspecific staining.

Figure S4: Localisation of Rab1A, Rab1B, Rab2, Rab4, Rab7 and Rab18. (A-F) Intracellular parasites expressing the indicated ddFKBPmyc-Rab-construct were grown for 18hrs in the presence of 1 µM Shld-1 prior to fixation. Co-expression of the Golgi marker GRASP-RFP, TgGalNac-YFP, the Golgi/ER marker TgERD-GFP, the Apicoplast marker FNR-RFP, or co-staining with α-proM2AP, or α-TgVP1 antibodies to label endosomal-like compartments (ELCs) were performed. The respective Rabs were detected with α-myc. Dapi is shown in blue. Scale bar: 5µm. Co-localisations were quantified by calculating the Pearson's correlation coefficient (R). Mean values and respective standard deviation of 10-16 parasites are indicated next to the respective image.

Figure S5: Localisation of Rab5A, Rab5B and Rab5C. (A, B, D) Intracellular parasites expressing indicated ddFKBPmyc-Rab5A, 5C and Rab5B-ddFKBPHA-construct were grown for 18hrs in presence of 1 µM Shld-1 prior to fixation. Co-expression of the Golgi marker GRASP-RFP, or co-staining with α-proM2AP, α-TgVP1or α-IMC was performed. To indicate the localisation of the respective Rab α-myc, or α-HA antibodies were used. Dapi is shown in blue. Scale bar: 5 µm. (C) Parasites co-expressing Ty-Rab5A and ddFKBPmyc-Rab5C were probed with α-Ty and α-myc antibodies. Rab5A and Rab5C show complete co-localisation. Scale bar: 5 µm. Co-localisation was quantified by calculating the Pearson‘s correlation coefficient (R). Mean values and respective standard deviation of 10-16 parasites are presented in a table beneath the respective image set.

Figure S6: Characterisation of Rab1A. (A) Western blot and immunofluorescence analysis of ddFKBPmyc-Rab1A(N126I) expressing parasites. For the western blot freshly lysed parasites were treated for 4hrs +/- 1 µM Shld-1 and for the immunofluorescence analysis intracellular parasites were treated for 18hrs +/- 1 µM Shld-1. Indicated antibodies were used. As an internal control for the western blot α-catalase antibodies were used. Dapi is shown in blue. Scale bar: 5 µm. (B) Growth analyses of the indicated parasite strains for 5 days +/- 1 µM Shld-1. The scale bar represents 1 mm. No significant growth defect was detected in parasites expressing ddFKBPmyc-Rab1A(N126I).

Figure S7: Characterisation of Rab7. (A, B) Western blot and immunofluorescence analyses of ddFKBPmyc-Rab7(G18E) and ddFKBPmyc-Rab7(N124I) expressing parasites. For the western blot freshly lysed parasites were treated for 4 h +/- 1 µM Shld-1 and for the immunofluorescence analyses intracellular parasites were treated for 18hrs +/- 1 µM Shld-1. Indicated antibodies were used. Dapi is shown in blue. As an internal control for the western blot α-catalase antibodies were used. Scale bar: 5 µm. **(**C) Growth analyses of parasites expressing indicated ddFKBPRab-constructs, which were inoculated on HFF cells and cultured for 5-6 days +/- Shld-1. The scale bar represents 1 mm. (D) Immunofluorescence analysis of intracellular parasites expressing ddFKBPmyc-Rab7(G18E) and wild type parasites RH $^{hxgprt-}$treated for 24hrs with 1µM Shld-1 and probed with indicated antibodies. Dapi is shown in blue. Scale bar: 5 µm. (E) Analysis of secretory organelles (MIC3, M2AP) and ELCs (CPL, VP1) in wildtype (RH $^{hxgprt-}$) and ddFKBPmyc-Rab7(G18E) expressing parasites using indicated antibodies. Parasites were grown in +/- 1 µM Shld-1 for 24hrs. Dapi is shown in blue. The scale bars represent 5µm.

Figure S8: Characterisation of Rab5B.

(A) Quantification of the localisation of rhoptry and microneme proteins in immunofluorescence analysis of parasites stably Rab5B-ddFKBPHA induced for 12, 18 and 24hrs with 1 µM Shld-1. 300-400 PVs of three independent experiments were analysed and normalised to RH $^{hxgprt-}$parasites. Average (AVG) and the respective standard deviation (STD) are presented. A tendency of MIC3 secretion after 24hrs post-induction with Shld-1 was detected, whereas M2AP, MIC2 and the rhoptry proteins ROP2-4 show no influence on the overexpression of Rab5B-ddFKBPHA. Fluorescence plus DIC images are shown (B) Western blot and immunofluorescence analysis of ddFKBPmyc-Rab5B(N152I) expressing parasites. For the western blot freshly lysed parasites were treated for 4hrs +/- 1 µM Shld-1 and for the immunofluorescence analysis intracellular parasites were treated for 18hrs +/- 1 µM Shld-1. Indicated antibodies were used. Dapi is shown in blue. As an internal control for the western blot α-catalase antibodies were used. Scale bar: 5 µm. (C) Growth analysis of the indicated parasite strains for 5 days in +/- 1 µM Shld-1. The scale bar represents 1 mm. (D) Immunofluorescence analysis of intracellular parasites expressing ddFKBPmyc-Rab5B(N152I) treated for 24hrs +/- 1 µM Shld-1 and immunolabelled with the indicated antibodies. The scale bars represent 5 µm.

Figure S9: Analysis of parasites overexpressing ddFKBPmyc-Rab5A and ddFKBPmyc-Rab5C. (A) Immunofluorescence analysis of intracellular parasites expressing ddFKBPmyc-Rab5A, ddFKBPmyc-Rab5C and wild type parasites RH $^{hxgprt-}$treated for 24hrs with 1 µM Shld-1 and probed with indicated antibodies (red) and Dapi (blue). For both overexpressors only MIC3, MIC8 and MIC11 are mislocalised. M2AP, MIC2 and AMA1 exhibit a normal apical localisation. (B) Replication assay of indicated parasites grown for 24hrs in presence, or absence of 1 µM Shld-1 prior to fixation. Average number of parasites per PV was determined. (C) Egress assay of indicated parasites grown for 36hrs +/- 1 µM Shld-1 before egress was triggered with A23187. Host cell lysis was determined 8 min after induction of egress and normalised with RH $^{hxgprt-}$parasites. For both overexpressors the egress is decreased. (D) Invasion assay of indicated parasites treated for 24hrs +/- 1 µM Shld-1, scratched and inoculated on fresh HFF cells. Subsequently invasion was determined and normalised to RH $^{hxgprt-}$parasites. (B-D) Mean values and the respective standard deviation of three independent experiments are presented (***indicates p-value of $P \leq 0.01$, **indicates $P \leq 0.02$ and *indicates $P \leq 0.07$ in a two tailed Student's test).

Figure S10: Expression of ddFKBPmyc-Rab5A(N158I) and ddFKBPmyc-Rab5C(N153I) results in a severe growth phenotype. (A, B) Western blot and immunofluorescence analyses of parasites expressing dominant negative versions of ddFKBPmyc-Rab5A(N158I) and ddFKBPmyc-Rab5C(N153I). For the western blot freshly lysed parasites were treated for 4hrs in presence (+), or absence (-) of 1 μM Shld-1 and for the immunofluorescence analysis intracellular parasites were treated for 18hrs +/- 1 μM Shld-1. The respective Rab protein was detected by α-ddFKBP antibodies (green). As an internal control for the western blot α-catalase antibodies were used. Dapi is shown in blue. Asterisk (*) indicates an unspecific signal in the western blot. The scale bars represent 5 μm. (C) Parasites (over)-expressing indicated versions of Rab5-GTPases were inoculated on HFF cells and incubated for 5-6 days +/-1 μM Shld-1. The scale bar represents 1 mm. Overexpression of Rab5A/C and expression of dominant negative versions results in severe growth defects.

Figure S11: Normal organelle formation and distribution in parasites expressing ddFKBPmyc-Rab5A(N158I). Immunofluorescence analysis of intracellular parasites stably expressing the dominant negative ddFKBPmyc-Rab5A(N158I) and wild type parasites RH $^{hxgprt-}$treated for 24hrs with 1 μM Shld-1 co-expressed with organellar markers for the apicoplast (FNR-RFP), the Golgi (GRASP-RFP), the Mitochondrion (HSP60-RFP), or co-stained with α-IMC (inner membrane complex) and α-GRA9 (dense granules) antibodies. To detect the expression of ddFKBPmyc-Rab5A(N158I) samples were additionally probed with α-myc antibodies. Dapi is stained in blue. Scale bars represent 5 μm. Expression of dominant negative ddFKBPmyc-Rab5A(N158I) shows no negative effects on all tested organelles.
